# Prolonged Edoxaban in Patients With Low Body Weight and Cancer-Associated Isolated Distal Deep Vein Thrombosis

**DOI:** 10.1016/j.jacadv.2025.101956

**Published:** 2025-07-04

**Authors:** Tomoyuki Nagai, Naohiko Nakanishi, Yugo Yamashita, Takeshi Morimoto, Nao Muraoka, Michihisa Umetsu, Yuji Nishimoto, Takuma Takada, Yoshito Ogihara, Tatsuya Nishikawa, Nobutaka Ikeda, Kazunori Otsui, Daisuke Sueta, Yukari Tsubata, Masaaki Shoji, Ayumi Shikama, Yutaka Hosoi, Yasuhiro Tanabe, Ryuki Chatani, Kengo Tsukahara, Kitae Kim, Satoshi Ikeda, Takeshi Kimura, Satoaki Matoba, the ONCO DVT Study Investigators

**Affiliations:** a Department of Cardiovascular Medicine, Graduate School of Medical Science, Kyoto Prefectural University of Medicine, Kamigyo-ku, Kyoto, Japan; b Department of Cardiovascular Medicine, Graduate School of Medicine, Kyoto University, Sakyo-ku, Kyoto, Japan; c Department of Clinical Epidemiology, Hyogo Medical University, Nishinomiya, Japan; d Division of Cardiology, Shizuoka Cancer Center, Nagaizumi-cho, Sunto-gun, Shizuoka, Japan; e Division of Vascular Surgery, Department of Surgery, Tohoku University Hospital, Aoba-ku, Sendai, Japan; f Division of Cardiology, Osaka General Medical Center, Sumiyoshi-ku, Osaka, Japan; g Department of Cardiology, Tokyo Women's Medical University, Shinjuku-ku, Tokyo, Japan; h Department of Cardiology and Nephrology, Mie University Graduate School of Medicine, Tsu, Japan; i Department of Onco-Cardiology, Osaka International Cancer Institute, Chuo-ku, Osaka-shi, Osaka, Japan; j Division of Cardiovascular Medicine, Toho University Ohashi Medical Center, Meguro-ku, Tokyo, Japan; k Department of General Internal Medicine, Kobe University Graduate School of Medicine, Chuo-ku, Kobe, Japan; l Department of Cardiovascular Medicine, Graduate School of Medical Sciences, Kumamoto University, Kumamoto, Japan; m Department of Internal Medicine, Division of Medical Oncology and Respiratory Medicine, Shimane University Faculty of Medicine, Izumo, Japan; n Department of Cardiovascular Medicine, National Cancer Center Hospital, Chuo-ku, Tokyo, Japan; o Department of Obstetrics and Gynecology, Faculty of Medicine, University of Tsukuba, Tsukuba, Japan; p Department of Cardiovascular Surgery, Kyorin University Faculty of Medicine, Mitaka-shi, Tokyo, Japan; q Department of Cardiology, St. Marianna University School of Medicine, Miyamae-ku, Kawasaki, Japan; r Department of Cardiovascular Medicine, Kurashiki Central Hospital, Kurashiki, Japan; s Division of Cardiology, Fujisawa City Hospital, Fujisawa, Japan; t Department of Cardiovascular Medicine, Kobe City Medical Center General Hospital, Chuo-ku, Kobe, Japan; u Department of Cardiovascular Medicine, Nagasaki University Graduate School of Biomedical Sciences, Nagasaki, Japan; v Department of Cardiology, Hirakata Kohsai Hospital, Hirakata, Japan

**Keywords:** cancer-associated thrombosis, edoxaban, isolated distal deep vein thrombosis, low body weight, venous thromboembolism

## Abstract

**Background:**

The ONCO DVT study revealed that 12-month edoxaban treatment for cancer-associated isolated distal deep vein thrombosis (IDDVT) was superior to 3-month edoxaban treatment. However, the influence of body weight on efficacy and safety remains unknown.

**Objectives:**

We compared 12-month and 3-month edoxaban treatments in patients with low body weight and cancer-associated IDDVT.

**Methods:**

In this prespecified subgroup analysis of the ONCO DVT study, we divided patients by body weight with a 60 kg cutoff. The primary endpoint was symptomatic recurrent venous thromboembolism or venous thromboembolism-related death at 12 months.

**Results:**

Of the 601 participants, 426 had low body weight, 99% receiving a reduced dose of edoxaban. The 1-year primary endpoint rate was significantly lower in the 12-month edoxaban group than in the 3-month group in both the low body weight (1.0% vs 6.2%, *P* = 0.003; OR: 0.15; 95% CI: 0.02-0.55) and the non-low body weight (1.0% vs 10.0%, *P* = 0.005; OR: 0.10; 95% CI: 0.01-0.54) subgroups. The 1-year major bleeding rate was not different between the 12-month and 3-month groups in the low body weight subgroup (7.0% vs 8.4%, *P* = 0.57), whereas in the non-low body weight subgroup, it was significantly higher in the 12-month edoxaban group than in the 3-month edoxaban group (14.7% vs 3.8%, *P* = 0.01).

**Conclusions:**

Twelve-month edoxaban treatment in cancer-associated IDDVT was superior to 3-month edoxaban treatment in terms of thrombotic events without increased bleeding risk among patients with low body weight but with increased bleeding risk among patients with non-low body weight.

Cancer is a significant risk factor for venous thromboembolism (VTE),^1^ which could be detrimental to the prognosis of patients with cancer. As advances in cancer treatment have increased the life expectancy of these patients, cancer-associated thrombosis has become an increasingly important global issue. A study reported that approximately 6.6% of patients with cancer had cancer-associated thrombosis and that isolated distal deep vein thrombosis (IDDVT) constituted 11% of these cases.^2^ Cancer-associated IDDVT has a not-low risk of VTE recurrence rate^3^^,^^4^ and progression to proximal deep vein thrombosis (DVT).^5^ Patients with cancer-associated IDDVT have high mortality, recurrence, and bleeding risk comparable to those with cancer-associated proximal DVT.^6^ The current guidelines have recommended prolonged anticoagulant therapy for patients with cancer-associated VTE in terms of prevention of thrombotic events.^7^^,^^8^ Nevertheless, the optimal anticoagulation duration for patients with cancer-associated IDDVT has not been established due to the difficulty in taking the good balance between thrombotic and bleeding risks.

Among patients with cancer, 34% were reported to have weight loss at the time of diagnosis.^9^ In the Hokusai VTE Cancer study, approximately 15% of patients with cancer had a body weight ≤60 kg.^10^ A meta-analysis evaluating direct oral anticoagulants (DOACs) for patients with atrial fibrillation or VTE demonstrated that patients with low body weight were associated with an increased risk of thromboembolism but have a comparable bleeding risk to those without low body weight.^11^ Whereas, active cancer could increase the risk of bleeding during anticoagulant therapy,^12^ and low body weight could have some concerns about an increased risk of bleeding with DOAC.^13^ However, there is limited data on the efficacy and safety of prolonged anticoagulation therapy in patients with low body weight and cancer-associated thrombosis.

The ONCO DVT study demonstrated that 12-month edoxaban treatment was superior in preventing symptomatic VTE recurrence or VTE-related death compared to the 3-month treatment, with a comparable risk of major bleeding between the 2 treatment groups.^14^ However, there could be some concerns about the application of these results for patients with low body weight. Thus, we conducted this prespecified subgroup analysis of the ONCO DVT study, focusing on patients with low body weight.

## Methods

### Study design and population

The ONCO DVT study was an investigator-initiated, multicenter, open-label, adjudicator-blinded randomized clinical trial conducted at 60 institutions in Japan, designed to compare 12-month and 3-month edoxaban treatment regimens in patients with cancer-associated IDDVT. Details of the ONCO-DVT study have been described previously.^14^ In brief, patients with active cancer who were newly diagnosed with IDDVT by ultrasonography were randomly assigned to either the 12-month or 3-month edoxaban treatment group in a 1-to-1 ratio and assessed for the superiority of long-term anticoagulation treatment with edoxaban. The detailed inclusion and exclusion criteria are provided in Supplemental Appendix 2.

**Figure 1:**
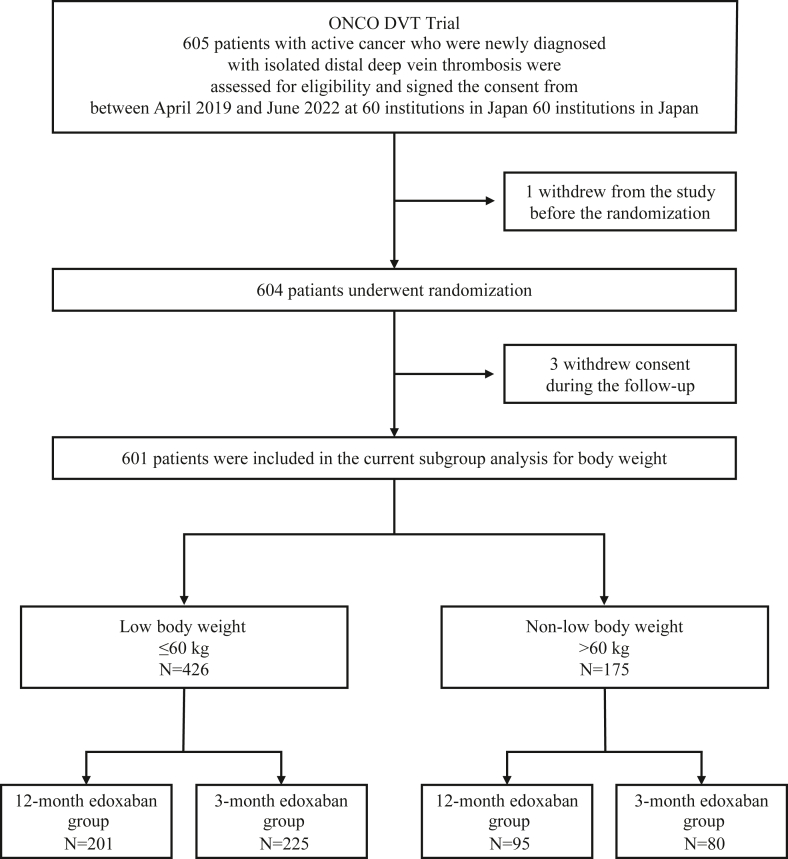
Study Flowchart The 601 patients in the intention-to-treat population of the ONCO DVT study (Edoxaban for 12 Months Versus 3 Months in Patients With Cancer With Isolated Distal Deep Vein Thrombosis) study were divided into 2 subgroups according to body weight with a cutoff of 60 kg.

In this prespecified subgroup analysis, we divided the entire cohort into 2 groups according to the body weight at diagnosis with a cutoff of 60 kg: low body weight group (≤60 kg) and non-low body weight group (>60 kg). The cutoff value of 60 kg was predetermined based on the dose reduction criteria of edoxaban for body weight. From April 2019 to June 2022, 605 patients were enrolled, and 604 patients were randomized. After excluding 3 patients who withdrew consent during follow-up, 601 patients in the intention-to-treat population were included in the current study (Figure 1).

### Patient characteristics and definitions

Active cancer was defined as cancer meeting one of the following criteria: newly diagnosed within 6 months of randomization; cancer treatment (such as surgery, chemotherapy, or radiotherapy) administered within 6 months of randomization; currently receiving cancer treatment; recurrence, local invasion, or distal metastases; and hematopoietic malignancy without complete remission. Definitions of the detailed baseline characteristics are provided in Supplemental Appendix 3.

### Treatment and follow-up

Edoxaban was administered orally at a fixed dose of 60 mg once daily (standard dose) or at a dose of 30 mg once daily (reduced dose) in patients with a creatinine clearance of 30 to 50 mL/min, a body weight of ≤60 kg, or receiving concomitant treatment with potent P-glycoprotein inhibitors. At the 3-month and 12-month visits after diagnosis, the patients underwent ultrasonography of the lower limb venous system and laboratory tests. During the follow-up period, the anticoagulation status and clinical events were recorded.

### Endpoints

The primary and secondary endpoints of the current study were identical to those used in the main analysis of the ONCO DVT study. The primary endpoint was a composite of symptomatic recurrent VTE and VTE-related death at 12 months. Symptomatic recurrent VTE was defined as new or recently worsening pulmonary embolism (PE) or DVT symptoms, new thrombi found on imaging tests, or worsening thrombi compared to the most recent image. Symptomatic recurrent VTE was not diagnosed if worsening of the thrombus was only apparent on imaging without worsening symptoms. Similarly, if a patient had a thrombus in the index vein with new symptoms, recurrent VTE was not considered to be present unless a thrombus extension was visible. VTE-related death was defined as death due to PE diagnosed at autopsy, death following clinically severe PE, or death unexplained by reasons other than PE.

The major secondary endpoint was major bleeding events at 12 months of treatment. Major bleeding was defined according to the International Society on Thrombosis and Haemostasis criteria, that is, fatal bleeding, symptomatic bleeding in a critical area or organ, and bleeding reducing hemoglobin levels ≥2 g/dL or requiring transfusions of ≥2 units of whole blood or red blood cells.^15^ Other secondary endpoints were symptomatic VTE recurrence, VTE-related death, asymptomatic recurrent VTE, all clinically relevant bleeding events, and all-cause death at 12 months. Clinically relevant bleeding events included major and nonmajor bleeding events. Clinically relevant nonmajor bleeding was defined as clinically overt bleeding (including bleeding detected only by imaging) that did not meet the criteria for major bleeding but led to physician-guided medical intervention, hospital admission, or further treatment for bleeding. Persistent edoxaban discontinuation was defined as discontinuation of edoxaban according to the study protocol or that lasting >14 days for any reason.

### Statistical analyses

Categorical variables are presented as numbers (percentages). Continuous variables are presented as mean ± SD or median (IQR), based on their distributions. Categorical variables were compared using the chi-square or Fisher exact tests. Continuous variables were compared using the Student’s *t*-test or the Wilcoxon rank-sum test. The cumulative incidence was estimated using the Kaplan-Meier method, and differences between the 12-month and 3-month edoxaban treatment groups were compared using the log-rank test. Furthermore, to take into account the competing risk of non-VTE-related death for the primary endpoint and all-cause death for the secondary endpoints, we used Gray method to estimate the cumulative incidence according to the Kaplan-Meier curves. We also calculated the ORs with the corresponding 95% CIs using logistic regression models, and the differences in the effects of the 12-month and 3-month edoxaban treatment regimens were evaluated according to the body weight subgroups using interaction terms in the models. We also performed per-protocol and as-treated analyses to avoid bias in an open-label-assigned group for sensitivity analysis (Supplemental Appendix 4). The analysis of the net adverse clinical events was performed in the intention-to-treat population. Net major adverse clinical events were a composite of symptomatic recurrent VTE, VTE-related death, or major bleeding. Net all adverse clinical events were a composite of symptomatic recurrent VTE, VTE-related death, asymptomatic recurrent VTE, or all clinically relevant bleeding. In addition, to investigate the influence of the edoxaban doses on clinical outcomes in the non-low body weight subgroup, we conducted a further subgroup analysis among the standard-dose and reduced-dose edoxaban subgroups. Reported *P* values were 2-tailed, and statistical significance was set at *P* < 0.05. The JMP version 18.1.0 software (SAS Institute Inc) was used for all analyses.

## Results

### Patient enrollment and characteristics

Of the 601 patients in the intention-to-treat population of the ONCO DVT study, 426 (70.9%) were assigned to the low body weight subgroup and 175 (29.1%) to the non-low body weight subgroup (Figure 1). The baseline patient characteristics between the low body weight and non-low body weight subgroups were presented in Table 1. Compared to the non-low body weight subgroup, patients in the low body weight subgroup were older (71.8 ± 9.7 years vs 68.4 ± 10.1 years, *P* < 0.001) and had a lower proportion of men (71 patients [17%] vs 96 patients [55%], *P* < 0.001). In the low body weight subgroup, the mean body weight was 49.6 ± 6.5 kg (men: 53.7 ± 5.4 kg, women: 48.7 ± 6.4 kg). A reduced dose of edoxaban 30 mg once daily was administered to 422 patients (99%) in the low body weight subgroup and 28 patients (16%) in the non-low body weight subgroup. No significant differences were noted in comorbidities between the 2 subgroups, except for the lower prevalence of hypertension and diabetes in the low body weight subgroup than in the non-low body weight subgroup. Patients with creatinine clearance <50 mL/min were more prevalent in the low body weight subgroup than in the non-low body weight subgroup (n = 118 [28%] vs n = 13 [7%]; *P* < 0.001). Clinical characteristics between the 12-month and 3-month edoxaban groups were well balanced in each subgroup (Supplemental Table 1). Details of cancer types were provided in Supplemental Table 2.

**Table 1:** Clinical Characteristics Compared Between Patients With and Without Low Body Weight

	Low Body Weight^a^ (N = 426)	Non-Low Body Weight^a^ (N = 175)	*P* Value
Baseline characteristics			
Age (y)	71.8 ± 9.7	68.4 ± 10.1	<0.001
Age ≥75 y, n (%)	189 (44)	56 (32)	0.006
Men, n (%)	71 (17)	96 (55)	<0.001
Body weight, kg	49.6 ± 6.5	70.1 ± 9.0	<0.001
Men	53.7 ± 5.4	69.4 ± 7.4	<0.001
Women	48.7 ± 6.4	70.8 ± 10.7	<0.001
Body mass index, kg/m^2^	20.9 ± 2.7	26.6 ± 4.0	<0.001
Men	20.2 ± 2.4	29.0 ± 4.1	<0.001
Women	21.0 ± 2.8	26.6 ± 4.1	<0.001
Symptoms at baseline, n (%)	87 (20)	35 (20)	1.00
Site of thrombosis, n (%)			
Bilateral, n (%)	164 (38)	59 (34)	0.16
Right side, n (%)	100 (23)	54 (31)	
Left side, n (%)	162 (38)	62 (35)	
Standard dose of edoxaban (60 mg per day), n (%)^b^	4 (1)	147 (84)	<0.001
Reduced dose of edoxaban (30 mg per day), n (%)^b^	422 (99)	28 (16)	<0.001
Cancer status			
Newly diagnosed cancer within 6 mos, n (%)	273 (64)	116 (66)	0.64
Chemotherapy administered within 6 mos, n (%)	200 (47)	83 (47)	0.93
Radiotherapy administered within 6 mos, n (%)	41 (10)	11 (6)	0.21
Recurrent cancer, n (%)	47 (11)	18 (10)	0.89
Metastatic disease, n (%)	107 (25)	40 (23)	0.60
ECOG performance status, n (%)^c^			
0	207 (49)	104 (59)	0.053
1	136 (32)	45 (26)	
≥2	83 (19)	26 (15)	
Comorbidities			
Hypertension, n (%)	169 (40)	94 (54)	0.002
Diabetes, n (%)	53 (12)	48 (27)	<0.001
Heart failure, n (%)	6 (1)	4 (2)	0.49
History of stroke, n (%)	17 (4)	10 (6)	0.39
History of venous thromboembolism, n (%)	20 (5)	13 (7)	0.24
History of major bleeding, n (%)	14 (3)	9 (5)	0.35
Transient risk factors for venous thromboembolism, n (%)^d^	112 (26)	39 (22)	0.35
Surgery within 2 months, n (%)	61 (14)	24 (14)	0.90
Laboratory results at diagnosis			
Creatinine clearance ≤50 mL/min, n (%)	118 (28)	13 (7)	<0.001
Anemia, n (%)^e^	299 (70)	103 (59)	0.010
Platelet count <100,000/μL, n (%)	19 (4)	12 (7)	0.23
D-dimer, μg/mL^f^	5.1 (2.3-11.3)	4.5 (2.1-9.9)	0.41
Concomitant medication			
Antiplatelet, n (%)	35 (8)	13 (7)	0.87
Steroids, n (%)	58 (14)	19 (11)	0.42
Statins, n (%)	93 (22)	41 (23)	0.67

Values are mean ± SD, median (IQR), or n (%).

ECOG = Eastern Cooperative Oncology Group.

a Low body weight was defined as a body weight ≤60 kg.

b Edoxaban was administered at a dose of 30 mg once daily (instead of 60 mg once daily) in patients with a creatinine clearance of 30 to 50 mL/min, body weight of ≤60 kg, or in those receiving concomitant treatment with potent P-glycoprotein inhibitors.

c ECOG performance status values range from 0 to 4, with higher values indicating greater disability.

d Transient risk factors for venous thromboembolism include recent surgery; recent immobilization; long-distance travel; central venous catheter use; pregnancy or puerperium; recent leg trauma, fractures, or burns; severe infection; and estrogen use.

e Anemia was diagnosed if the hemoglobin level was <13 g/dL for men and <12 g/dL for women.

f Data were missing for 12 patients in the low body weight subgroup and 22 patients in the non-low body weight subgroup.

### Edoxaban treatment

**Figure 2:**
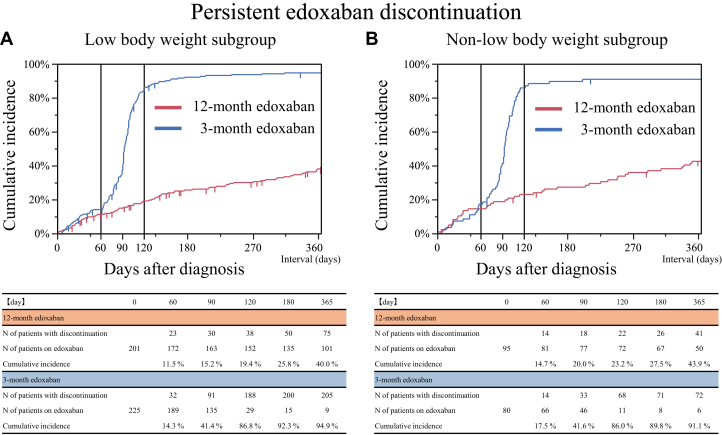
Time-to-Event Curves of Persistent Edoxaban Discontinuation Persistent edoxaban discontinuation was defined as discontinuation according to the study protocol or lasting >14 days for any reason. (A) Low body weight subgroup. (B) Non-low body weight subgroup.

In the low body weight subgroup, the cumulative 120-day incidence of persistent edoxaban discontinuation was 19.4% in the 12-month edoxaban group and 86.8% in the 3-month group (Figure 2A). In the non-low body weight subgroup, the cumulative 120-day incidence of persistent edoxaban discontinuation was 23.2% in the 12-month group and 86.0% in the 3-month group (Figure 2B). The reasons for persistent discontinuation of edoxaban are detailed in Supplemental Table 3. In both the low body weight and non-low body weight subgroups, common reasons for persistent discontinuation of edoxaban in the 12-month edoxaban group included bleeding events and cancer progression, whereas per-protocol discontinuation was more prevalent in the 3-month edoxaban group.

### Primary endpoint

**Figure 3:**
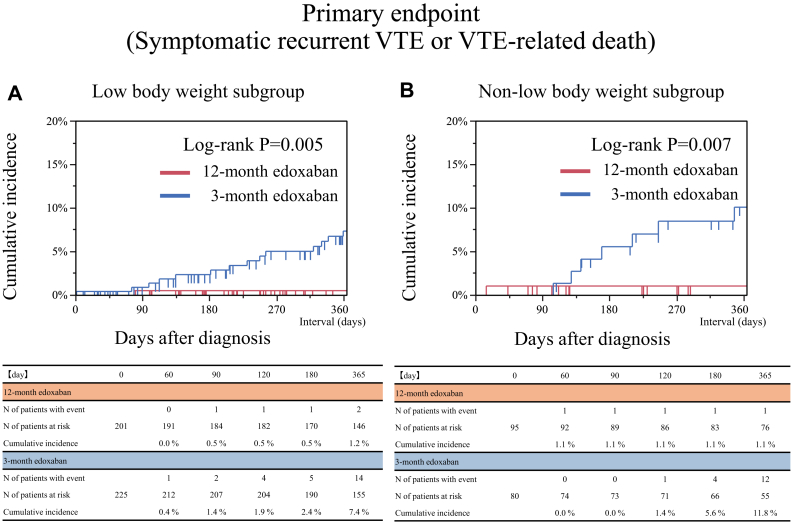
Time-to-Event Curves of the Primary Endpoint of Symptomatic VTE Recurrence or VTE-Related Death (A) Low body weight subgroup. (B) Non-low body weight subgroup. VTE = venous thromboembolism.

**Figure 4:**
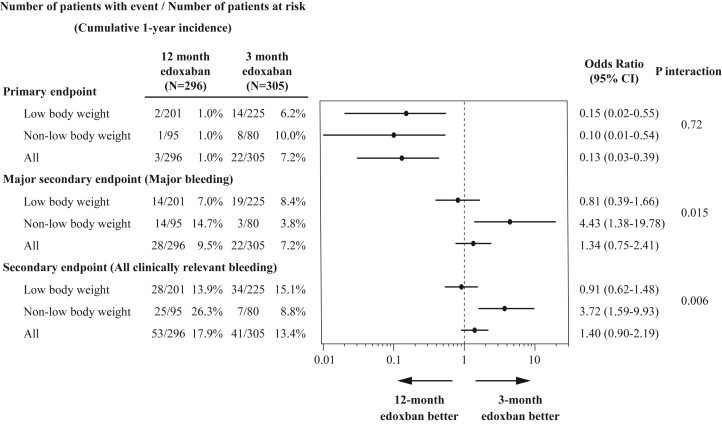
Subgroup Analyses for Body Weight The ORs for the clinical endpoints were described according to the body weight subgroups.

In both the low body weight and non-low body weight subgroups, the 1-year rate of the primary endpoint (a composite of symptomatic recurrent VTE and VTE-related death) at 12 months was significantly lower in the 12-month edoxaban group than that in the 3-month group (low body weight: 1.0% vs 6.2%, *P* = 0.003, and non-low body weight: 1.0% vs 10.0%, *P* = 0.005) (Table 2, Figures 3A and 3B). Even after taking into account the competing risk of non-VTE-related death for the primary endpoint, the results were consistent with the main analysis (Supplemental Figure 1). In both subgroups, a lower risk in the 12-month edoxaban group relative to the 3-month group was significant for the primary endpoint than the 3-month edoxaban group (low body weight: OR: 0.15; 95% CI: 0.02-0.55; and non-low body weight: OR: 0.10; 95% CI: 0.01-0.54) (Table 2). No significant interaction between body weight and treatment groups was noted for the primary endpoint (interaction *P* = 0.72) (Figure 4).

**Table 2:** Clinical Outcomes at 12 Months

	Low Body Weight				Non-Low Body Weight			
	12-Month Edoxaban Group (N = 201)	3-Month Edoxaban Group (N = 225)	OR (95% CI)	*P* Value	12-Month Edoxaban Group (N = 95)	3-Month Edoxaban Group (N = 80)	OR (95% CI)	*P* Value
Primary endpoint								
Symptomatic recurrent venous thromboembolism or venous thromboembolism-related death, n (%)	2 (1.0)	14 (6.2)	0.15 (0.02-0.55)	0.003	1 (1.0)	8 (10.0)	0.10 (0.01-0.54)	0.005
Major secondary endpoint								
Major bleeding, n (%)^a^	14 (7.0)	19 (8.4)	0.81 (0.39-1.66)	0.57	14 (14.7)	3 (3.8)	4.43 (1.38-19.78)	0.011
Other secondary endpoints								
Symptomatic venous thromboembolism recurrence events, n (%)	2 (1.0)	14 (6.2)	0.15 (0.02-0.55)	0.003	1 (1.0)	8 (10.0)	0.10 (0.01-0.54)	0.005
Venous thromboembolism-related deaths, n (%)^b^	0 (0)	0 (0)	-	-	0 (0)	0 (0)	-	-
New or worsening thrombus images in any imaging tests during follow-up without any symptoms, n (%)^c^	11 (5.5)	35 (15.6)	0.31 (0.15-0.62)	0.001	12 (12.6)	11 (13.8)	0.91 (0.37-2.21)	0.83
All clinically relevant bleeding events, n (%)^d^	28 (13.9)	34 (15.1)	0.91 (0.53-1.56)	0.73	25 (26.3)	7 (8.8)	3.72 (1.59-9.83)	0.002
Deaths from any causes, n (%)	51 (25.4)	59 (26.2)	0.96 (0.62-1.48)	0.84	15 (15.8)	18 (22.5)	0.65 (0.30-1.38)	0.26

Analyses were performed for the full analysis set based on the intention-to-treat approach, which included all patients who had undergone randomization after excluding those who withdrew their consent. For patients who did not experience an event, the time to the first event was censored at day 365 or the last day the patient had a complete assessment of study outcomes, whichever came first. We calculated the ORs, computed using a logistic regression model, along with the corresponding 95% CIs, for all clinical endpoints, which were not adjusted for multiple comparisons.

a Major and nonmajor bleeding events were classified according to the International Society on Thrombosis and Haemostasis criteria.

b Death due to pulmonary embolism diagnosed prior to death or at autopsy, or death unexplained by other than pulmonary embolism.

c Appearance of new or worsening thrombus images in the pulmonary arteries and deep veins on imaging tests (ultrasonography of lower limb venous system, computed tomography examination, pulmonary perfusion scintigraphy, pulmonary angiography, and venography) that did not match the definition of a symptomatic venous thromboembolism recurrence and were not associated with new or worsening symptoms.

d For patients with more than 1 event, only the first event was counted.

### Major secondary endpoint: major bleeding

**Figure 5:**
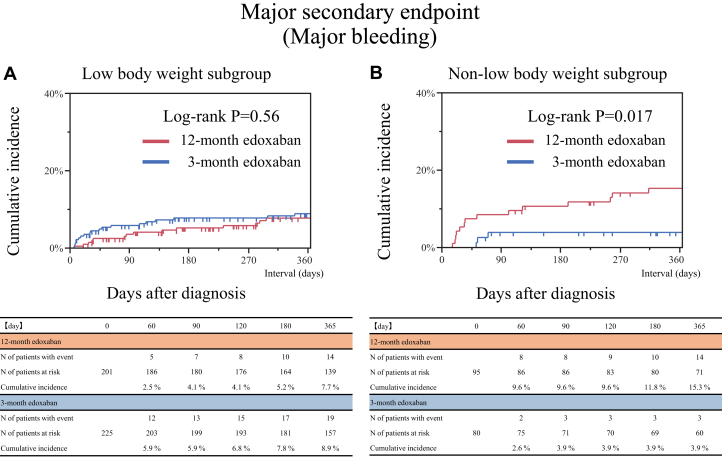
Time-to-Event Curves of the Major Secondary Endpoint of Major Bleeding Major bleeding was defined according to International Society on Thrombosis and Haemostasis criteria. (A) Low body weight subgroup. (B) Non-low body weight subgroup.

In the low body weight subgroup, no significant difference was noted in the 1-year rate of the major secondary endpoint (major bleeding) between the 12-month and 3-month edoxaban groups (7.0% vs 8.4%, *P* = 0.57) (Table 2, Figure 5A), while in the non-low body weight subgroup, it was significantly higher in the 12-month edoxaban group than in the 3-month edoxaban group (14.7% vs 3.8%, *P* = 0.011) (Table 2, Figure 5B). Even after taking into account the competing risk of all-cause death for the major secondary endpoints, the results were consistent with the main analysis (Supplemental Figure 2). No significant difference was noted in the risk of major bleeding between the 12-month and 3-month edoxaban groups in the low body weight subgroup (OR: 0.81; 95% CI: 0.39-1.66), while in the non-low body weight subgroup, the 12-month edoxaban group had a significantly higher risk of major bleeding compared to that of the 3-month edoxaban group (OR: 4.43; 95% CI: 1.38-19.78) (Table 2). Significant interaction between body weight and treatment groups was noted regarding the major secondary endpoint (interaction *P* = 0.015) (Figure 4). Details of the bleeding sites for the major secondary endpoint are provided in Supplemental Table 4.

### Other secondary endpoints

**Figure 6:**
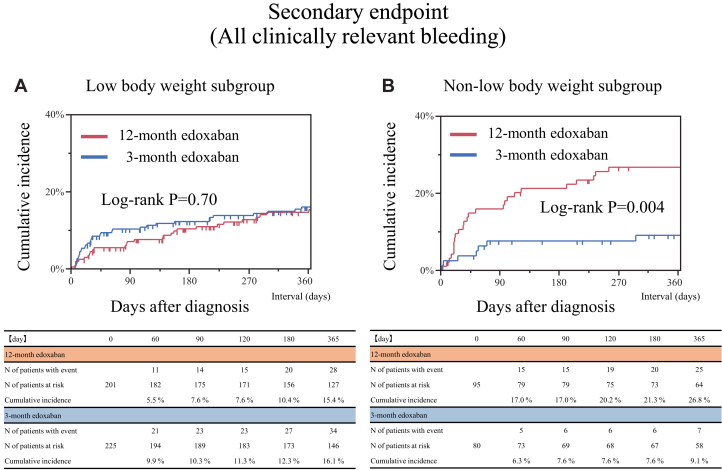
Time-to-Event Curves of the Secondary Endpoint of All Clinically Relevant Bleeding (A) Low body weight subgroup. (B) Non-low body weight subgroup.

No significant difference was noted in the 1-year rate of all clinically relevant bleeding events between the 12-month and 3-month edoxaban treatment groups in the low body weight subgroup (13.9% vs 15.1%, *P* = 0.73) (Table 2, Figure 6A), while in the non-low body weight subgroup, it was significantly higher in the 12-month edoxaban group than in the 3-month group (26.3% vs 8.8%, *P* = 0.002) (Table 2, Figure 6B). Even after taking into account the competing risk of all-cause death for all clinically relevant bleeding events, the results were consistent with the main analysis (Supplemental Figure 3). No significant difference was noted in the risk of all clinically relevant bleeding between the 12-month and 3-month edoxaban groups in the low body weight subgroup (OR: 0.91; 95% CI: 0.53-1.56), while the higher risk of the 12-month edoxaban group relative to the 3-month edoxaban group was significant for all clinically relevant bleeding in the non-low body weight subgroup (OR: 3.72; 95% CI: 1.59-9.83). There was a significant interaction between the body weight and treatment groups regarding all clinically relevant bleeding events (interaction *P* = 0.006) (Figure 4).

### Sensitivity analyses, net adverse clinical events analyses, and additional subgroup analysis

The results of the per-protocol and as-treated analyses were consistent with those of the primary analysis (Supplemental Tables 5 and 6, Supplemental Figures 4 to 13). In the analyses of net adverse clinical events with a composite of thrombotic and bleeding events, the net clinical benefit of the 12-month over 3-month edoxaban treatment was significant in the low body weight subgroup (7.5% vs 13.8%; OR: 0.50; 95% CI: 0.26-0.95). However, the net clinical benefit in the 12-month edoxaban group as compared to the 3-month edoxaban group was attenuated in the non-low body weight subgroup (15.8% vs 13.8%; OR: 1.18; 95% CI: 0.51-2.79) (Supplemental Table 7). The subgroup analysis focusing on antiplatelet therapy or gastrointestinal cancers that are considered to have a higher risk of bleeding was shown in Supplemental Tables 8 and 9. In the low-body-weight subgroup of patients who received antiplatelet therapy or had gastrointestinal cancers, no significant increase in the risk of major bleeding or all clinically relevant bleeding were observed in the 12-month edoxaban treatment group compared to the 3-month edoxaban treatment group.

Further subgroup analysis according to the doses of edoxaban among the non-low body weight subgroup was presented in Supplemental Table 10 and Supplemental Figures 14 to 17. The 12-month edoxaban treatment group exhibited higher 1-year rates of major bleeding and all clinically relevant bleeding than the 3-month edoxaban treatment group among patients with an edoxaban dose of 60 mg (major bleeding: 14.1% vs 4.3%, *P* = 0.038; all clinically relevant bleeding: 26.9% vs 8.7%, *P* = 0.003). Whereas, no significant difference was noted in the 1-year rates of all clinically relevant bleeding between the 12-month and 3-month edoxaban groups among patients with an edoxaban dose of 30 mg (23.5% vs 9.1%, *P* = 0.31) (Supplemental Table 10).

## Discussion

In this study, we demonstrated that, even in patients with low body weight and cancer-associated IDDVT, the 12-month edoxaban treatment was superior to the 3-month treatment in terms of the composite outcome of symptomatic VTE recurrence or VTE-related death. In addition, prolonged 12-month edoxaban treatment did not increase the risk of bleeding events compared with that of short-term 3-month edoxaban treatment in patients with low body weight and cancer-associated IDDVT. However, in patients with non-low body weight receiving standard-dose edoxaban, the 12-month edoxaban treatment exhibited higher rates of major bleeding and all clinically relevant bleeding than the 3-month edoxaban treatment (Central Illustration).

**Central Illustration:**
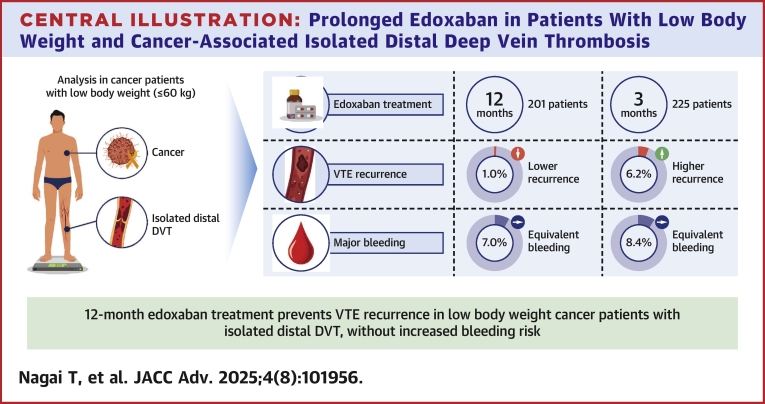
Prolonged Edoxaban in Patients With Low Body Weight and Cancer-Associated Isolated Distal Deep Vein Thrombosis DVT = deep vein thrombosis; VTE = venous thromboembolism.

Cancer-associated VTE carries a higher risk of bleeding during anticoagulation treatment than that of VTE in patients without cancer.^4^ Additionally, cancer-associated VTE is associated with an increased risk of recurrence.^16^ Cancer-associated IDDVT might present a lower risk of fatal PE than proximal DVT.^3^ However, cancer-associated IDDVT might be considered a precursor to proximal DVT.^5^ Consequently, prolonged anticoagulation therapy could have a potential benefit in preventing thrombotic events in these patients with cancer-associated thrombosis.^6^ However, it would be crucial to carefully weigh the benefits and risks of extended anticoagulation therapy.^17^, ^18^, ^19^

Weight loss is common in patients with cancer.^8^ The Hokusai VTE Cancer study reported that 15.9% of patients in the edoxaban group had low body weight (≤60 kg).^9^ Within this low body weight subgroup, the rate of VTE recurrence was comparable between the edoxaban and dalteparin groups; however, a trend toward a higher incidence of major bleeding was noted in the edoxaban group. Additionally, Asians have a higher risk of bleeding than non-Asians.^20^ In the ONCO DVT study conducted in Japan, a relatively high proportion of patients with cancer had low body weight.^14^ In this subgroup analysis focusing on low body weight, the low body weight subgroup showed a high-bleeding risk profile including high proportions of older women and reduced creatinine clearance. Notably, almost all patients in the low body weight subgroup received a reduced dose of edoxaban according to the dose adjustment criteria. Our results demonstrated that prolonged 12-month edoxaban treatment was effective and well tolerated, even in patients with low body weight, suggesting a potential benefit of prolonged anticoagulation therapy for patients with low body weight and cancer-associated IDDVT.

Recently, several reports have demonstrated the efficacy and safety of DOACs in patients with low body weight. In the subgroup analysis of the ENGAGE AF-TIMI 48 trial, plasma edoxaban concentration was consistent across extremes of body weight, and patients with low body weight under 55 kg demonstrated comparable efficacy and favorable bleeding risk compared to that with warfarin.^21^ A retrospective cohort study found that the use of DOACs is associated with a lower risk of any bleeding event than that of warfarin in patients with low body weight and nonvalvular atrial fibrillation.^22^ Additionally, the benefit of super-low-dose edoxaban (15 mg once daily) has been reported in very old patients with low body weight and atrial fibrillation.^23^ However, these studies were not limited to patients with cancer, who have a high risk of both thrombosis and bleeding. In patients with low body weight and cancer-associated thrombosis, assessment of additional risk factors for bleeding, such as renal/liver dysfunction, has been recommended before commencing DOAC treatment.^24^ In the subanalysis of the ONCO DVT study focusing on frailty factors such as chronic renal dysfunction and anemia, patients with chronic renal dysfunction or anemia experienced fewer thrombotic recurrences without an elevated risk of bleeding in patients with 12-month edoxaban treatment.^25^^,^^26^ Due to the limited data for the use of DOACs in patients with low body weight and cancer, the efficacy and safety of long-term DOAC use in these patients has not yet been established. Our study demonstrated that 12-month edoxaban treatment prevented VTE recurrence in low-body-weight patients with cancer-associated IDDVT, with no significant increased risk of bleeding. The dose of edoxaban is adjusted based on a body weight threshold of 60 kg; therefore, almost all patients with low body weight in our study received a reduced dose of edoxaban. Edoxaban, with its weight-based dose reduction criteria, might be preferable for patients with low body weight and cancer-associated IDDVT that could theoretically have a lower risk of thrombosis than proximal VTE.

In the non-low body weight subgroup, the 12-month edoxaban treatment was associated with a reduction in the thrombotic events compared to that of the 3-month edoxaban treatment. However, the bleeding events increased with the 12-month edoxaban treatment compared with those of the 3-month edoxaban treatment. As a note, a high proportion of patients in the non-low body weight subgroup received a standard dose of edoxaban (60 mg). While the use of low-dose edoxaban is relatively uncommon in Western populations,^27^ Asians, who often have a higher proportion of low body weight, tend to use low-dose edoxaban more frequently.^28^ Another subgroup analysis of the ONCO DVT study focusing on edoxaban doses reported that 12-month edoxaban treatment consistently demonstrated superiority over the 3-month regimen in reducing thrombotic risk in the standard-dose and reduced-dose edoxaban subgroups.^29^ Conversely, while the 12-month edoxaban treatment increased the risk of major bleeding compared with that of the 3-month edoxaban treatment in the standard-dose edoxaban group, the cumulative incidence of major bleeding was comparable between the 12-month and 3-month treatments in the reduced-dose edoxaban group. Consistent with these findings, our results also demonstrated that the standard-dose group had lower VTE recurrence but increased bleeding with the 12-month edoxaban treatment, whereas the reduced-dose edoxaban group did not show an increased risk of bleeding events with the 12-month edoxaban treatment in the non-low body weight subgroup. Further clinical trials would be warranted to investigate the optimal dosing of edoxaban in these patients.

### Study Limitations

This study has several limitations. First, the ONCO DVT study was an open-label trial, which could have introduced bias. However, all clinical endpoints were assessed by community members who were unaware of the group to which the participants were assigned. In addition, to mitigate the influence of diagnostic testing strategies on the primary endpoint, the ONCO DVT trial included symptomatic recurrent VTE as the primary endpoint, not asymptomatic recurrent VTE. Second, a relatively small number of participants completed the edoxaban treatment protocol throughout the study. In the 12-month edoxaban treatment group, some patients prematurely discontinued treatment because of bleeding or cancer progression. Third, the sample size of each group may have been insufficient for a robust subgroup analysis. Although 28 patients (16%) in the non-low body weight group received 30 mg of edoxaban, the reasons for dose reduction were not collected. Prolonged edoxaban treatment did not decrease the primary endpoint in patients who received 30 mg edoxaban in the non-low body weight subgroup; however, the statistical power was insufficient to draw definitive conclusions. In addition, because edoxaban treatment includes body weight as a dose reduction criterion, it was difficult to perform a weight-specific analysis using edoxaban dose as a confounder. Thus, the current results should be regarded as exploratory. Fourth, the ONCO DVT study was conducted in Japan, where a larger proportion of participants received reduced doses of edoxaban. Previous reports in patients with cancer-related VTE have indicated that reduced doses of edoxaban were more commonly used in Asian patients than in Western patients.^25^^,^^26^ Additionally, East Asian populations exhibit an increased risk of bleeding during antithrombotic therapies compared to those of Caucasian patients.^20^ Therefore, caution would be needed when generalizing these findings outside Japan.

## Conclusions

Twelve-month edoxaban treatment in cancer-associated IDDVT was superior to 3-month edoxaban treatment in terms of thrombotic events without increased bleeding risk among patients with low body weight but with increased bleeding risk among patients with non-low body weight.

Perspectives **COMPETENCY IN MEDICAL KNOWLEDGE:** The 12-month edoxaban treatment is effective and well tolerated, when compared to the 3-month edoxaban treatment, for patients with low body weight and cancer-associated isolated DVT. However, the 12-month edoxaban treatment had a higher bleeding rate than did the 3-month edoxaban treatment in patients without low body weight with cancer-associated isolated DVT, which may be associated with the edoxaban dose.

**TRANSLATIONAL OUTLOOK:** Future research should determine which patients with cancer should be considered for prolonged edoxaban treatment and the optimal edoxaban dose for cancer-associated IDDVT.

## Funding support and author disclosures

Funding was provided by 10.13039/501100022274Daiichi Sankyo Co, Ltd (Tokyo, Japan), which had no role in the study design, data collection, analysis, and interpretation, or the writing of the report. Dr Yamashita received lecture fees from Bayer Healthcare, 10.13039/100002491Bristol-Myers Squibb, 10.13039/100004319Pfizer, and 10.13039/501100022274Daiichi Sankyo, and grant support from 10.13039/501100000801Bayer Healthcare and 10.13039/501100022274Daiichi Sankyo. Dr Morimoto reports lecturer fees from 10.13039/100004325AstraZeneca, Bristol Myers Squibb, 10.13039/501100022274Daiichi Sankyo, 10.13039/100015643Japan Lifeline, 10.13039/100015993Kowa, 10.13039/100004319Pfizer, and 10.13039/501100013420Tsumura; article fees from Bristol Myers Squibb and 10.13039/100004319Pfizer; and membership on advisory boards for Novartis and Teijin. Dr Nishimoto has received lecture fees from Bayer Healthcare, Bristol Myers Squibb, 10.13039/100004319Pfizer, and 10.13039/501100022274Daiichi Sankyo. Dr Ogihara received lecture fees from Bayer Healthcare, Bristol Myers Squibb, 10.13039/100004319Pfizer, and 10.13039/501100022274Daiichi Sankyo, and research funds from Bayer Healthcare and 10.13039/501100022274Daiichi Sankyo. Dr N. Ikeda received lecture fees from Bayer Healthcare, Bristol Myers Squibb, and 10.13039/501100022274Daiichi Sankyo. Dr Tsubata received lecture fees from AstraZeneca, Chugai Pharmaceutical, Bristol Myers Squibb, Kyowa Kirin, 10.13039/100004319Pfizer, Taiho Pharmaceutical, Takeda Pharmaceutical, and 10.13039/501100022274Daiichi Sankyo, and grant support from 10.13039/501100022274Daiichi Sankyo, AstraZeneca, and Ono Pharmaceutical. Dr S. Ikeda received lecture fees from Bayer Healthcare, Bristol Myers Squibb, and 10.13039/501100022274Daiichi Sankyo. Dr Mo received lecture fees from Bayer Healthcare. All other authors have reported that they have no relationships relevant to the contents of this paper to disclose.
