# Supplementary material for: Prolonged Edoxaban in Patients With Low Body Weight and Cancer-Associated Isolated Distal Deep Vein Thrombosis
Source: JACC Adv. 2025 Jul 4;4(8):101956. doi: 10.1016/j.jacadv.2025.101956 (PMC12271068; doi:10.1016/j.jacadv.2025.101956)
Supplement: Supplemental Material [file mmc1.docx]

**Supplemental** **Appendix**

##

## **Supplemental Appendix 1: Participating Centers**

Department of Cardiovascular Medicine, Kyoto University Hospital (Yugo YAMASHITA); Department of Onco-Cardiology, Osaka International Cancer Institute (Masafumi FUJITA); Department of Cardiovascular Medicine, Saiseikai Noe Hospital (Ichiro KOUCHI); Department of Cardiology, Osaka Red Cross Hospital (Tsukasa INADA); Department of Cardiovascular Medicine, Japanese Red Cross Otsu Hospital (Kazuaki KAITANI); Department of Cardiovascular Medicine, Kakogawa Central City Clinics (Hiroaki NAKAMURA); Department of Cardiovascular Medicine, Cancer Institute Hospital (Taro SHIGA); Department of Vascular Surgery, Kansai Medical University Medical Center (Nobuko YAMAMOTO); Department of Cardiovascular Medicine, University Hospital Kyoto Prefectural University of Medicine (Satoaki MATOBA); Department of Cardiovascular Surgery, Kyorin University Faculty of Medicine (Yutaka HOSOI); Department of Cardiovascular Medicine, Kindai University Hospital (Gaku NAKAZAWA); Department of Cardiovascular Medicine, Kumamoto University Hospital (Daisuke SUETA); Department of Cardiovascular Medicine, Kurashiki Central Hospital (Kazushige KADOTA); Department of Cardiovascular Surgery, Kurume University Hospital (Shinichi HIROMATSU); Department of Cardiovascular Medicine, Kuwana City Medical Center (Norikazu YAMADA); Department of Cardiovascular Medicine, Gunma University (Norimichi KOITABASHI); Department of Cardiovascular Medicine, Kobe City Medical Center General Hospital (Yutaka FURUKAWA); Department of General Internal Medicine, Kobe University Hospital (Kazunori OTSUI); Department of Cardiovascular Medicine, Kohka Public Hospital (Tomohiro DOUKA); Department of Cardiovascular Surgery, Fukushima Medical University Hospital (Daiki WAKAMATSU); Department of Cardiovascular Medicine, Kokura Memorial Hospital (Kenji ANDO); Department of General Internal Medicine / Department of Cardiovascular Medicine, National Cancer Center Hospital (Masaaki SHOJI); Department of Cardiovascular Medicine, NHO Okayama Medical Center (Hiroto SHIMOKAWAHARA); Department of Cardiovascular Medicine, NHO Kyoto Medical Center (Kosuke DOI); Department of Cardiovascular Medicine, Saiseikai Yokohamashi Nanbu Hospital (Tsutomu ENDO); Department of Cardiovascular Surgery, Saiseikai Wakayama Hospital (Atsutoshi HATADA); Department of Cardiovascular Medicine, Saku Central Hospital Advanced Care Center (Yoshikazu YAZAKI); Department of Cardiovascular Medicine, Shiga General Hospital (Takeshi UENO); Department of Cardiovascular Medicine, Shizuoka Cancer Center (Nao MURAOKA); Department of Cardiovascular Medicine, Shizuoka City Shizuoka Hospital (Ryuzo NAWATA); Department of Respiratory Medicine and Clinical Oncology, Shimane University Hospital (Yukari TSUBATA); Department of Cardiovascular Medicine, Shimada General Medical Center (Yoshiaki TSUYUKI); Department of Cardiology, St. Marianna University School of Medicine (Yasuhiro TANABE); Department of Cardiovascular Medicine, Medical Research Institute Kitano Hospital (Moriaki INOKO); Department of Obstetrics and Gynecology, University of Tsukuba Hospital (Toyomi SATO); Department of Cardiovascular Medicine, Tenri Hospital (Toshihiro TAMURA); Department of Cardiovascular Medicine, Tokyo Women’s Medical University Hospital (Yuichiro MINAMI); Department of Cardiovascular Medicine, Tokyo Metropolitan Tama Medical Center (Hiroyuki TANAKA); Department of Cardiovascular Medicine, Toho University Ohashi Medical Center (Nobutaka IKEDA); Department of Cardiovascular Medicine, Toho University Omori Medical Center (Shinji HISATAKE); Department of General Surgery, Tohoku University Hospital (Hisashi GOTO); Department of Cardiovascular Medicine, Nagasaki University Hospital (Koji MAEMURA); Department of Obstetrics and Gynecology, Nara Medical University Hospital (Ryuji KAWAGUCHI); Department of Cardiovascular Intensive Care, Nippon Medical School Hospital (Tsuyoshi YAMAMOTO); Department of Cardiovascular Medicine, Japanese Red Cross Wakayama Medical Center (Shojiro TATSUSHIMA); Department of Cardiovascular Medicine, Hyogo Prefectural Amagasaki General Medical Center (Yukihiro SATO); Department of Cardiovascular Medicine, Hirakata Kohsai Hospital (Shoji KITAGUCHI); Department of Cardiovascular Medicine, Fukui Prefectural Hospital (Susumu FUJINO); Department of Vascular Surgery, Saiseikai Yahata General Hospital (Shinsuke MII); Department of Cardiovascular Medicine, Fujisawa City Hospital (Kengo TSUKAHARA); Department of Cardiovascular Medicine, Makiminato Central Hospital (Naoya MAEHIRA); Department of Cardiovascular Medicine, Mie University Hospital (Kaoru DOHI); Department of Cardiovascular Medicine, Mitsubishi Kyoto Hospital (Takafumi YOKOMATSU); Department of Cardiovascular Medicine, Japanese Red Cross Musashino Hospital (Takashi ASHIKAGA); Department of Cardiovascular Surgery, Yokohama Minami Kyousai Hospital (Makoto MO); Hospital Department of Cardiovascular Medicine, Yokohama Rosai Hospital (Kazuhiko YUMOTO); Department of Vascular Surgery, Rakuwakai Otowa Hospital (Ryoji TAKEDA); Department of Cardiovascular Medicine, Niigata University Graduate School of Medicine and Dentistry (Shinya FUJIKI); Department of Internal Medicine, Niigata Cancer Center Niigata Hospital (Yuji OKURA); Department of Surgery of the Lower Gastrointestinal Surgery, Hyogo College of Medicine (Jihyung SONG).

## **Supplemental Appendix 2: Inclusion Criteria and Exclusion Criteria**

**Inclusion criteria**

Patients with active cancer who are newly diagnosed with isolated distal deep vein thrombosis (DVT) by ultrasonography of the lower limb vein system, are scheduled for treatment with anticoagulation therapy, and satisfy all the following inclusion criteria:

1. Male or female patients aged 20 years or older

2. Patients who have provided informed consent

3. Patients with a new diagnosis of DVT that is objectively confirmed by ultrasonography extended to the whole deep venous system of both legs (either symptomatic or asymptomatic)

4. Patients with active cancer* at randomization

5. Patients scheduled for DVT treatment or recurrence prevention with anticoagulation therapy

Note: Cancer in this study includes all malignant tumors, including epithelial carcinomas, sarcomas, and hematologic malignancies such as leukemia.

Note: Active cancer satisfies one of the following criteria: 1. Newly diagnosed with cancer within 6 months of randomization. 2. Cancer treatment (surgery, chemotherapy, radiotherapy, etc.) performed within 6 months of randomization. 3. Currently receiving cancer treatment (surgery, chemotherapy, radiotherapy, etc.). 4. Has recurrence, local invasion, or distant metastases. 5. Patients with a hematopoietic malignancy who have not achieved complete remission.

Note: Ultrasonography of the lower limb vein system: All patients are evaluated by whole leg ultrasonography using a high-frequency (5-10MHz) linear probe. The examination observes the veins from the inferior vena cava to the ankle level with the patient in a supine position. In addition to imaging diagnostics using a color Doppler, the compression method will also be used to check for thrombi. If evaluation with the patient in a supine position is insufficient for examination of the veins of the lower limbs, then the examination may be conducted with the patient in a seated position with their feet on the floor, at the discretion of the expert sonographer. The diagnostic criteria for isolated distal DVT are the presence of thrombi or lack of blood flow with distal compression, in addition to non-collapse with compression.

**Exclusion criteria**

Patients who meet any of the following criteria are excluded:

1. Patients who started anticoagulation therapy (heparin, fondaparinux, warfarin, direct oral anticoagulant [DOAC], etc.) for the index event 10 or more days before randomization

2. Patients currently on oral anticoagulation therapy (warfarin, DOAC) for treatment / prevention of recurrence of venous thromboembolism (VTE) for purposes other than the index event; or treated with oral anticoagulation therapy (warfarin, DOAC) for indications other than VTE at the time of the diagnosis

3. Patients treated with thrombolysis therapy or an inferior vena cava filter at the index event

4. Patients with creatinine clearance less than 30 ml/min

5. Patients who are expected to have a life prognosis of 3 months or less

6. Patients with pulmonary embolism (PE) (symptomatic or asymptomatic)

7. Patients who are regarded as not appropriate for participation in the study by the attending physician

## **Supplemental Appendix 3:** **Definition of the baseline characteristics**

Eastern Cooperative Oncology Group (ECOG) performance status (PS): 0. Fully active, at pre-disease performance levels without restriction. 1. Restricted physically strenuous activity, but ambulatory and able to carry out work of a light and sedentary nature. 2. Ambulatory and capable of all self-care but unable to carry out any work activities. Up and about more than 50% of waking hours. 3. Capable of only limited self-care, confined to bed or chair more than 50% of waking hours. 4. Completely disabled. Cannot carry on any self-care. Totally confined to bed or a chair. Diabetes: Blood glucose level ≥200 mg/dl 2 or more hours after loading in a glucose tolerance test, casual blood glucose ≥200 mg/dl, fasting blood glucose ≥126 mg/dl, or Hemoglobin A1c ≥6.5%. Even when the above tests are not performed diabetes is defined if the patient has already been clinically diagnosed with diabetes or is taking medication to treat diabetes. Heart failure: Cases that satisfy any of the following criteria are defined as heart failure: history of a hospitalization for heart failure, clinical heart failure symptoms at a New York Heart Association II (can walk on flat ground but cannot jog) or higher or left ventricular ejection fraction <40%. History of major bleeding: Cases that satisfy any of the following criteria are defined as having a history of major bleeding: a history of bleeding into vital organs, history of bleeding that required a blood transfusion, history of bleeding with a reduction in the hemoglobin of ≥2 g/dl, or a history of bleeding that required fluid transfusion, vasopressors, or surgical treatment. Transient risk factors for venous thromboembolism included recent surgery, recent immobilization, long-distance travel, central venous catheter use, pregnancy or puerperium, recent leg trauma, fracture or burn, severe infection, and estrogen use. Anemia was diagnosed if the value of hemoglobin was <13 g/dL for men and <12 g/dL for women.

## **Supplemental Appendix 4: Analysis populations**

<Full analysis set>

All patients randomly assigned to the treatment group will be included, inclusive of all data obtained from first day of the group assignment until the end of the follow-up period.

<Per-protocol analysis>

Patients who were randomly assigned to and administered the study drug (edoxaban) at least once, with no major deviations from the research protocol, will be included, and data from the day of assignment to the end the follow-up period will be included. We defined 3-month edoxaban group as patients who did not receive edoxaban at 120 days after diagnosis who assigned to 3-month edoxaban group, and 12-month edoxaban group as patients who received edoxaban at 120 days after diagnosis who assigned to 12-month edoxaban group. We excluded patients enrolled with exclusion criteria at randomization, patients lost to follow-up before 120 days after diagnosis, and patients who died before 120 days after diagnosis.

<As-treated analysis>

Patients who were randomly assigned to and administered the study drug (edoxaban) at least once will be included, inclusive of all data obtained from the day of assignment until the end of the follow-up period. Regardless of randomly assigned group, 1) the patients who did not receive edoxaban at 120 days after diagnosis were defined as short edoxaban group, and 2) the patients who received edoxaban at 120 days after diagnosis were defined as long edoxaban group. We excluded patients enrolled with exclusion criteria at randomization, patients lost to follow-up before 120 days after diagnosis, and patients who died before 120 days after diagnosis.

# **Supplemental Tables**

## **Supplemental Table 1: Clinical characteristics compared between 12- and 3-month edoxaban group in the low body weight and non- low body weight subgroups.**

|  | **Low body weight subgroup ^a^** | | | **Non-low body weight subgroup ^a^** | | |
| --- | --- | --- | --- | --- | --- | --- |
|  | **12-month edoxaban**  **(N=201)** | **3-month edoxaban**  **(N=225)** | **P-value** | **12-month edoxaban**  **(N=95)** | **3-month edoxaban**  **(N=80)** | **P value** |
| **Baseline characteristics** |  |  |  |  |  |  |
| Age (years) | 73.2±8.7 | 70.6±10.3 | 0.024 | 68.4±10.0 | 68.5±10.1 | 0.80 |
| Age ≥75 years, No. (%) | 101 (50) | 88 (39) | 0.024 | 30 (32) | 26 (33) | 1.00 |
| Men, No. (%) | 39 (19) | 32 (14) | 0.16 | 55 (58) | 41 (51) | 0.45 |
| Body weight, kg | 49.6±6.4 | 46.5±6.7 | 0.83 | 70.3±8.9 | 69.9±9.2 | 0.69 |
| Men | 52.4±6.0 | 55.2±4.3 | 0.043 | 70.1±7.8 | 68.7±6.9 | 0.47 |
| Women | 49.0±6.3 | 48.5±6.5 | 0.49 | 70.5±10.4 | 71.1±11.1 | 0.86 |
| Body mass index, kg/m^2^ | 20.9±2.7 | 20.8±2.7 | 0.57 | 26.4±3.8 | 26.7±4.2 | 0.78 |
| Men | 19.7±2.5 | 20.8±2.1 | 0.043 | 24.8±2.6 | 24.2±2.7 | 0.32 |
| Women | 21.2±2.7 | 20.8±2.8 | 0.12 | 28.7±4.1 | 29.3±4.1 | 0.42 |
| Symptoms at baseline, No. (%) | 33 (16) | 54 (24) | 0.055 | 20 (21) | 15 (19) | 0.85 |
| Site of thrombosis, No. (%) |  |  |  |  |  |  |
| Bilateral, No. (%) | 84 (42) | 80 (36) | 0.39 | 34 (36) | 25 (31) | 0.71 |
| Right side, No. (%) | 46 (23) | 54 (24) |  | 27 (28) | 27 (34) |  |
| Left side, No. (%) | 71 (35) | 91 (40) |  | 34 (36) | 28 (35) |  |
| Standard dose of edoxaban (60 mg per day), No. (%) ^b^ | 2 (1) | 2 (1) | 1.00 | 78 (82) | 69 (86) | 0.54 |
| Reduced dose of edoxaban (30 mg per day), No. (%) ^b^ | 199 (99) | 223 (99) | 1.00 | 17 (18) | 11 (14) | 0.54 |
| **Cancer status** |  |  |  |  |  |  |
| Newly diagnosed cancer within 6 months, No. (%) | 122 (61) | 151 (67) | 0.19 | 62 (65) | 54 (68) | 0.87 |
| Chemotherapy administered within 6 months, No. (%) | 98 (49) | 102 (45) | 0.50 | 44 (46) | 39 (49) | 0.76 |
| Radiotherapy administered within 6 months, No. (%) | 16 (8) | 25 (11) | 0.32 | 4 (4) | 7 (9) | 0.35 |
| Recurrent cancer, No. (%) | 23 (11) | 24 (11) | 0.88 | 8 (8) | 10 (13) | 0.46 |
| Metastatic disease, No. (%) | 49 (24) | 58 (26) | 0.82 | 18 (19) | 22 (28) | 0.21 |
| ECOG performance status, No. (%) ^c^ |  |  |  |  |  |  |
| 0 | 100 (50) | 107 (48) | 0.38 | 61 (64) | 43 (54) | 0.28 |
| 1 | 58 (29) | 78 (35) |  | 20 (21) | 25 (31) |  |
| ≥2 | 43 (21) | 40 (18) |  | 14 (15) | 12 (15) |  |
| **Comorbidities** |  |  |  |  |  |  |
| Hypertension, No. (%) | 79 (39) | 90 (40) | 0.92 | 54 (57) | 40 (50) | 0.45 |
| Diabetes, No. (%) | 27 (13) | 26 (12) | 0.56 | 27 (28) | 21 (26) | 0.87 |
| Heart failure, No. (%) | 3 (1) | 3(1) | 1.00 | 4 (4) | 0 (0) | 0.13 |
| History of stroke, No. (%) | 9 (4) | 8 (4) | 0.63 | 5 (5) | 5 (6) | 1.00 |
| History of venous thromboembolism, No. (%) | 12 (6) | 8 (4) | 0.26 | 8 (8) | 5 (6) | 0.77 |
| History of major bleeding, No. (%) | 5 (2) | 9 (4) | 0.43 | 2 (2) | 7 (9) | 0.082 |
| Transient risk factors for venous thromboembolism, No. (%) ^d^ | 58 (29) | 54 (24) | 0.27 | 22 (23) | 17 (21) | 0.86 |
| Surgery within 2 months, No. (%) | 32 (16) | 29 (13) | 0.41 | 9 (9) | 15 (19) | 0.083 |
| **Laboratory results at diagnosis** |  |  |  |  |  |  |
| Creatinine clearance ≤50 mL/min, No. (%) | 59 (29) | 59 (26) | 0.52 | 10 (11) | 3 (4) | 0.15 |
| Anemia, No. (%) ^e^ | 146 (73) | 153 (68) | 0.34 | 53 (56) | 50 (63) | 0.44 |
| Platelet count <100,000/μL, No. (%) | 7 (3) | 12 (5) | 0.48 | 5 (5) | 7 (9) | 0.39 |
| D-dimer, μg/mL ^f^ | 5.1 (2.2-10.7) | 5.1 (2.3-12.5) | 0.79 | 5.3 (2.0-11.9) | 3.7 (2.1-9.9) | 0.44 |
| **Concomitant medication** |  |  |  |  |  |  |
| Antiplatelet, No. (%) | 18 (9) | 17 (8) | 0.60 | 9 (9) | 4 (5) | 0.39 |
| Steroids, No. (%) | 27 (13) | 31 (14) | 1.00 | 7 (7) | 12 (15) | 0.14 |
| Statins, No. (%) | 48 (24) | 45 (20) | 0.35 | 23 (24) | 18 (23) | 0.86 |

Data are presented as mean ± standard deviation, median (interquartile range), or as numbers and percentages.

^a^ Low body weight was defined as a body weight ≤60 kg.

^b^ Edoxaban was administered at a dose of 30 mg once daily (instead of 60 mg once daily) in patients with a creatinine clearance of 30 to 50 ml/min, body weight of ≤60 kg, or in those receiving concomitant treatment with potent P-glycoprotein inhibitors.

^c^ Eastern Cooperative Oncology Group (ECOG) performance status values range 0–4, with higher values indicating greater disability.

^d^ Transient risk factors for venous thromboembolism include recent surgery; recent immobilization; long-distance travel; central venous catheter use; pregnancy or puerperium; recent leg trauma, fractures, or burns; severe infection; and estrogen use.

^e^ Anemia was diagnosed if the hemoglobin level was <13 g/dL for men and <12 g/dL for women.

^f^ In the low body weight subgroup, data were missing for 12 patients in the 3-month edoxaban group and 10 patients in the 12-month edoxaban group. In the non-low body weight subgroup, data were missing for 7 patients in the 3-month edoxaban group and 5 patients in the 12-month edoxaban group.

## **Supplemental Table 2: Types of cancer at baseline**

| **Types of cancer,**  **No. (%)** | **Low body weight** | | **Non-low body weight** | |
| --- | --- | --- | --- | --- |
|  | **12-month edoxaban**  **(N=201)** | **3-month edoxaban**  **(N=225)** | **12-month edoxaban**  **(N=95)** | **3-month edoxaban**  **(N=80)** |
| Ovary | 31 | 41 | 11 | 7 |
| Uterus | 29 | 28 | 8 | 16 |
| Lung | 26 | 29 | 12 | 5 |
| Colon | 15 | 27 | 12 | 4 |
| Pancreas | 17 | 20 | 8 | 1 |
| Stomach | 9 | 13 | 6 | 5 |
| Blood | 9 | 15 | 6 | 3 |
| Breast | 15 | 9 | 6 | 3 |
| Bladder | 3 | 9 | 8 | 3 |
| Kidney/ureter | 6 | 3 | 0 | 4 |
| Skin | 3 | 1 | 1 | 5 |
| Prostate | 4 | 4 | 3 | 1 |
| Liver | 4 | 3 | 1 | 2 |
| Brain | 7 | 2 | 0 | 2 |
| Esophagus | 3 | 1 | 4 | 3 |
| Gall bladder/bile duct | 4 | 2 | 0 | 0 |
| Thyroid gland | 0 | 0 | 1 | 0 |
| Multiple | 6 | 3 | 1 | 2 |
| Others | 10 | 15 | 7 | 7 |

Types of cancer at the baseline are described in the intention-to-treat population.

## **Supplemental Table 3: Reasons for persistent edoxaban discontinuation**

| **Reasons, No. (%)** | **Low body weight** | | **Non-low body weight** | |
| --- | --- | --- | --- | --- |
|  | **12-month edoxaban**  **(N=75/201)** | **3-month edoxaban**  **(N=205/225)** | **12-month edoxaban**  **(N=41/95)** | **3-month edoxaban**  **(N=72/80)** |
| Per-protocol discontinuation ^a^ | 0 (0) | 160 (78) | 0 (0) | 56 (78) |
| Due to bleeding events | 14 (19) | 18 (9) | 16 (39) | 3 (4) |
| Due to drug side effect | 7 (9) | 4 (2) | 3 (7) | 1 (1) |
| Due to cancer progression | 23 (31) | 3 (1) | 6 (15) | 6 (8) |
| Patient’s decision | 10 (11) | 3 (1) | 4 (10) | 0 (0) |
| Physician’s decision | 6 (8) | 0 (0) | 3 (7) | 0 (0) |
| Others | 15 (20) | 17 (8) | 9 (22) | 6 (8) |

Reasons for persistent edoxaban discontinuation were described in the intention-to-treat population.

^a^ Per-protocol discontinuation was defined as a discontinuation if the patients in the 3-month edoxaban treatment group stopped edoxaban 3 months after the diagnosis (≥61 days to <120 days) according to the study protocol.

##

## **Supplemental Table 4: Bleeding sites of major bleeding**

|  | **Low body weight** | | **Non-low body weight** | |
| --- | --- | --- | --- | --- |
|  | 12-month edoxaban  (N=201) | 3-month edoxaban  (N=225) | 12-month edoxaban  (N=95) | 3-month edoxaban  (N=80) |
| Major Bleeding | 14 | 19 | 14 | 3 |
| Sites of Bleeding, N (%) |  |  |  |  |
| Intra-cranial | 1 (7.1) | 1 (5.3) | 2 (1.4) | 0 (0) |
| Trachea | 0 (0) | 1 (5.3) | 0 (0) | 0 (0) |
| Gastrointestinal (Upper) | 2 (14.3) | 6 (31.6) | 2 (1.4) | 0 (0) |
| Gastrointestinal (Lower) | 8 (57.1) | 7 (36.8) | 7 (50.0) | 2 (66.7) |
| Urinary tract | 0 (0) | 3 (15.8) | 1 (7.1) | 0 (0) |
| Body cavity | 1 (7.1) | 0 (0) | 0 (0) | 0 (0) |
| Genital | 0 (0) | 1 (5.3) | 0 (0) | 0 (0) |
| Subcutaneous | 0 (0) | 0 (0) | 1 (7.1) | 0 (0) |
| Other sites | 2 (14.3) | 0 (0) | 1 (7.1) | 1 (33.3) |

## **Supplemental Table 5: Clinical outcomes at 12 months (per-protocol analysis)**

|  | **Low body weight** | | | | **Non-low body weight** | | | |
| --- | --- | --- | --- | --- | --- | --- | --- | --- |
|  | **12-month edoxaban group**  **(N=151)** | **3-month edoxaban group**  **(N=173)** | **Odds ratio**  **(95% CI)** | **P value** | **12-month edoxaban group**  **(N=72)** | **3-month edoxaban group**  **(N=61)** | **Odds ratio**  **(95% CI)** | **P value** |
| **Primary endpoint** |  |  |  |  |  |  |  |  |
| Symptomatic recurrent venous thromboembolism or venous thromboembolism-related death, No. (%) | 1(0.7) | 11 (6.4) | 0.10 (0.01–0.51) | 0.003 | 0 (0) | 8 (13.1) | - | - |
| **Major secondary endpoint** |  |  |  |  |  |  |  |  |
| Major bleeding, No. (%) ^a^ | 7 (4.6) | 10 (5.8) | 0.79 (0.28–2.12) | 0.97 | 5 (6.9) | 2 (3.3) | 2.20 (0.46–15.77) | 0.34 |
| **Other secondary endpoints** |  |  |  |  |  |  |  |  |
| Symptomatic venous thromboembolism recurrence events, No. (%) | 1 (0.7) | 11 (6.4) | 0.10 (0.01–0.51) | 0.003 | 0 (0) | 8 (13.1) | - | - |
| Venous thromboembolism-related deaths, No. (%) ^b^ | 0 (0) | 0 (0) | - | - | 0 (0) | 0 (0) | - | - |
| New or worsening thrombus images in any imaging tests during follow-up without any symptoms, No. (%) ^c^ | 8 (5.3) | 30 (17.3) | 0.27(0.11–0.58) | < 0.001 | 8 (11.1) | 10 (16.4) | 0.64 (0.23–1.73) | 0.38 |
|  |  |  |  |  |  |  |  |  |
| All clinically relevant bleeding events, No. (%) ^d^ | 16 (10.6) | 24 (13.9) | 0.74 (0.37–1.43) | 0.37 | 10 (13.9) | 5 (8.2) | 1.81 (0.60–6.09) | 0.30 |
| Deaths from any causes, No. (%) | 22 (14.6) | 31 (17.9) | 0.78(0.43–1.41) | 0.96 | 5 (6.9) | 9 (14.8) | 0.43(0.13–1.33) | 0.14 |

Per-protocol analysis included those patients who were randomized, and administered the study drug (edoxaban) at least once, with no major deviations from the research protocol. Data from the day of assignment to the end the follow-up period were included. We defined the 3-month edoxaban group as patients who did not receive edoxaban at 120 days after the diagnosis who were assigned to the 3-month edoxaban group, and 12-month edoxaban group as those patients who received edoxaban at 120 days after the diagnosis and who were assigned to the 12-month edoxaban group. We excluded patients enrolled with exclusion criteria at randomization, patients lost to follow-up before 120 days after diagnosis, and patients who died before 120 days after diagnosis. The 95% confidence intervals have not been adjusted for multiple comparisons.

## **Supplemental Table 6: Clinical outcomes at 12 months (As-treated analysis)**

|  | **Low body weight** | | | | **Non-low body weight** | | | |
| --- | --- | --- | --- | --- | --- | --- | --- | --- |
|  | **12-month edoxaban group**  **(N=180)** | **3-month edoxaban group**  **(N=203)** | **Odds ratio**  **(95% CI)** | **P value** | **12-month edoxaban group**  **(N=83)** | **3-month edoxaban group**  **(N=76)** | **Odds ratio**  **(95% CI)** | **P value** |
| **Primary endpoint** |  |  |  |  |  |  |  |  |
| Symptomatic recurrent venous thromboembolism or venous thromboembolism-related death, No. (%) | 1 (0.6) | 11 (5.9) | 0.09 (0.004–0.458) | 0.002 | 0 (0) | 9 (11.8) | - | - |
| **Major secondary endpoint** |  |  |  |  |  |  |  |  |
| Major bleeding, No. (%) ^a^ | 11 (6.1) | 15 (7.4) | 0.82 (0.36–1.81) | 0.62 | 5 (6.0) | 6 (7.9) | 0.75 (0.21–2.59) | 0.64 |
| **Other secondary endpoints** |  |  |  |  |  |  |  |  |
| Symptomatic venous thromboembolism recurrence events, No. (%) | 1 (0.6) | 11 (5.9) | 0.09 (0.004–0.458) | 0.002 | 0 (0) | 9 (11.8) | - | - |
| Venous thromboembolism-related deaths, No. (%) ^b^ | 0 (0) | 0 (0) | - | - | 0 (0) | 0 (0) | - | - |
| New or worsening thrombus images in any imaging tests during follow-up without any symptoms, No. (%) ^c^ | 13 (7.2) | 33 (16.3) | 0.40 (0.20–0.77) | 0.006 | 9 (10.8) | 14 (18.4) | 0.54 (0.21–1.31) | 0.17 |
|  |  |  |  |  |  |  |  |  |
| All clinically relevant bleeding events, No. (%) ^d^ | 21 (11.7) | 32 (15.8) | 0.71 (0.39–1.27) | 0.24 | 10 (12.0) | 13 (17.1) | 0.66 (0.27–1.61) | 0.37 |
| Deaths from all causes, No. (%) | 28 (15.6) | 42 (20.7) | 0.71 (0.41–1.19) | 0.19 | 6 (7.2) | 13 (17.1) | 0.38 (0.13–1.01) | 0.053 |

As-treated analysis included those patients who were randomized and administered the study drug (edoxaban) at least once, with no major deviations from the research protocol. Data from the day of assignment to the end the follow-up period were included. We defined the 3-month edoxaban group as patients who did not receive edoxaban at 120 days after the diagnosis who were assigned to the 3-month edoxaban group, and 12-month edoxaban group as those patients who received edoxaban at 120 days after the diagnosis and who were assigned to the 12-month edoxaban group. We excluded patients enrolled with exclusion criteria at randomization, patients lost to follow-up before 120 days after diagnosis, and patients who died before 120 days after diagnosis. The 95% confidence intervals have not been adjusted for multiple comparisons.

^a^ Major and nonmajor bleeding events were classified according to the criteria of the International Society on Thrombosis and Hemostasis.

^b^ Death due to pulmonary embolism diagnosed prior to death or at autopsy, or death unexplained by other than pulmonary embolism.

^c^ Appearance of new or worsening thrombus images in the pulmonary arteries and deep veins on imaging tests (ultrasonography of lower limb venous system, computed tomography examination, pulmonary perfusion scintigraphy, pulmonary angiography, venography) that do not match the definition of symptomatic venous thromboembolism recurrence and are not associated with new or worsening symptoms.

^d^ For patients who had more than one event, only the first was counted.

## **Supplemental Table 7: Net adverse clinical events at 12 months**

| **Net major adverse clinical events** ^a^ | |  |  |  |
| --- | --- | --- | --- | --- |
|  | **12-month edoxaban  (N=296)** | **3-month edoxaban  (N=305)** | **Absolute difference  (95%CI)** | **Odds ratio  (95%CI)** |
| Low body weight | 15/201 (7.5%) | 31/225 (13.8%) | -6.3% (-12.1% to -0.5%) | 0.50 (0.26–0.95) |
| Non- low body weight | 15/95 (15.8%) | 11/80 (13.8%) | 2.0% (-8.0% to 12.6%) | 1.18 (0.51–2.79) |
| **Net all adverse clinical events** ^b^ | |  |  |  |
|  | **12-month edoxaban  (N=296)** | **3-month edoxaban  (N=305)** | **Absolute difference  (95%CI)** | **Odds ratio  (95%CI)** |
| Low body weight | 37/201 (18.4%) | 73/225 (32.4%) | -14.0% (-22.2% to -5.9%) | 0.47 (0.30–0.73) |
| Non- low body weight | 32/95 (33.7%) | 22/80 (27.5%) | 6.2% (-7.5% to 19.8%) | 1.34 (0.70–2.59) |

^a^ Net major adverse clinical events were a composite of symptomatic recurrent VTE, VTE-related death, or major bleeding. ^b^ Net all adverse clinical events were a composite of symptomatic recurrent VTE, VTE-related death, asymptomatic recurrent VTE, or all clinically relevant bleeding.

## **Supplemental Table 8: Clinical outcomes at 12 months in the antiplatelet therapy subgroup**

|  | **Low body weight** | | | | **Non-low body weight** | | | |
| --- | --- | --- | --- | --- | --- | --- | --- | --- |
|  | **12-month edoxaban group**  **(N=18)** | **3-month edoxaban group**  **(N=17)** | **Odds ratio**  **(95% CI)** | **P value** | **12-month edoxaban group**  **(N=9)** | **3-month edoxaban group**  **(N=4)** | **Odds ratio**  **(95% CI)** | **P value** |
| **Primary endpoint** |  |  |  |  |  |  |  |  |
| Symptomatic recurrent venous thromboembolism or venous thromboembolism-related death, No. (%) | 0 (0) | 0 (0) | - | - | 0 (0) | 1 (25.0) | - | 0.11 |
| **Major secondary endpoint** |  |  |  |  |  |  |  |  |
| Major bleeding, No. (%) ^a^ | 2 (11.1) | 1 (5.9) | 1.67 (0.38–7.29) | 0.50 | 2 (22.2) | 0 (0.0) | 1.67 (0.38–7.29) | 0.50 |
| **Other secondary endpoints** |  |  |  |  |  |  |  |  |
| Symptomatic venous thromboembolism recurrence events, No. (%) | 0 (0) | 0 (0) | - | - | 0 (0) | 1 (25.0) | - | 0.11 |
| Venous thromboembolism-related deaths, No. (%) ^b^ | 0 (0) | 0 (0) | - | - | 0 (0) | 0 (0) | - | - |
| New or worsening thrombus images in any imaging tests during follow-up without any symptoms, No. (%) ^c^ | 1 (11.1) | 1 (5.9) | 0.94 (0.04–25.1) | 0.97 | 0 (0) | 1 (25.0) | - | 0.11 |
|  |  |  |  |  |  |  |  |  |
| All clinically relevant bleeding events, No. (%) ^d^ | 4 (22.2) | 3 (17.6) | 1.10 (0.40–3.07) | 0.85 | 3 (33.3) | 0 (0) | 1.10 (0.40–3.07) | 0.85 |
| Deaths from all causes, No. (%) | 7 (38.9) | 8 (47.1) | 0.72 (0.18–2.75) | 0.63 | 1 (11.1) | 0 (0) | - | 0.38 |

The analyses were performed for the full analysis set based on the intention-to-treat approach, which included all the patients who had undergone randomization after excluding patients who withdrew consent. For patients who did not experience an event, the time to the first event was to be censored at day 365, or the last day the patient had a complete assessment for study outcomes, whichever came first. We calculated the odds ratios, computed using the logistic regression model along with the corresponding 95% confidence intervals for all clinical endpoints, which have not been adjusted for multiple comparisons.

^a^ Major and nonmajor bleeding events were classified according to the criteria of the International Society on Thrombosis and Hemostasis.

^b^ Death due to pulmonary embolism diagnosed prior to death or at autopsy, or death unexplained by other than pulmonary embolism.

^c^ Appearance of new or worsening thrombus images in the pulmonary arteries and deep veins on imaging tests (ultrasonography of lower limb venous system, computed tomography examination, pulmonary perfusion scintigraphy, pulmonary angiography, venography) that do not match the definition of a symptomatic venous thromboembolism recurrence and are not associated with new or worsening symptoms.

^d^ For patients who had more than one event, only the first was counted.

## **Supplemental Table 9: Clinical outcomes at 12 months in the gastrointestinal cancer subgroup**

|  | **Low body weight** | | | | **Non-low body weight** | | | |
| --- | --- | --- | --- | --- | --- | --- | --- | --- |
|  | **12-month edoxaban group**  **(N=52)** | **3-month edoxaban group**  **(N=66)** | **Odds ratio**  **(95% CI)** | **P value** | **12-month edoxaban group**  **(N=31)** | **3-month edoxaban group**  **(N=22)** | **Odds ratio**  **(95% CI)** | **P value** |
| **Primary endpoint** |  |  |  |  |  |  |  |  |
| Symptomatic recurrent venous thromboembolism or venous thromboembolism-related death, No. (%) | 0 (0) | 3 (4.5) | - | 0.060 | 0 (0) | 2 (9.1) | - | 0.057- |
| **Major secondary endpoint** |  |  |  |  |  |  |  |  |
| Major bleeding, No. (%) ^a^ | 3 (5.8) | 7 (10.6) | 0.52 (0.11–1.96) | 0.34 | 5 16.1) | 0 (0) | - | 0.017 |
| **Other secondary endpoints** |  |  |  |  |  |  |  |  |
| Symptomatic venous thromboembolism recurrence events, No. (%) | 0 (0) | 3 (4.5) | 0.09 (0.004–0.458) | 0.060 | 0 (0) | 2 (9.1) | - | 0.057 |
| Venous thromboembolism-related deaths, No. (%) ^b^ | 0 (0) | 0 (0) | - | - | 0 (0) | 0 (0) | - | - |
| New or worsening thrombus images in any imaging tests during follow-up without any symptoms, No. (%) ^c^ | 2 (3.8) | 10 (15.2) | 0.22 (0.03–0.90) | 0.034 | 1 (3.2) | 4 (18.2) | 0.15 (0.01–1.11) | 0.064 |
|  |  |  |  |  |  |  |  |  |
| All clinically relevant bleeding events, No. (%) ^d^ | 5 (9.6) | 8 (12.1) | 0.77 (0.22–2.47) | 0.67 | 9 (29.0) | 0 (0) | - | 0.001 |
| Deaths from all causes, No. (%) | 16 (30.8) | 28 (42.2) | 0.60 (0.28–1.29) | 0.19 | 5 (16.1) | 4 (18.2) | 0.87 (0.20–3.92) | 0.845 |

The analyses were performed for the full analysis set based on the intention-to-treat approach, which included all the patients who had undergone randomization after excluding patients who withdrew consent. For patients who did not experience an event, the time to the first event was to be censored at day 365, or the last day the patient had a complete assessment for study outcomes, whichever came first. We calculated the odds ratios, computed using the logistic regression model along with the corresponding 95% confidence intervals for all clinical endpoints, which have not been adjusted for multiple comparisons.

^a^ Major and nonmajor bleeding events were classified according to the criteria of the International Society on Thrombosis and Hemostasis.

^b^ Death due to pulmonary embolism diagnosed prior to death or at autopsy, or death unexplained by other than pulmonary embolism.

^c^ Appearance of new or worsening thrombus images in the pulmonary arteries and deep veins on imaging tests (ultrasonography of lower limb venous system, computed tomography examination, pulmonary perfusion scintigraphy, pulmonary angiography, venography) that do not match the definition of a symptomatic venous thromboembolism recurrence and are not associated with new or worsening symptoms.

^d^ For patients who had more than one event, only the first was counted.

## **Supplemental Table 10: Clinical outcomes at 12 months in the non-low body weight subgroup (divided by edoxaban dose)**

|  | **Standard dose edoxaban (60 mg)** | | | | **Reduced dose edoxaban (30 mg)** | | | |
| --- | --- | --- | --- | --- | --- | --- | --- | --- |
|  | **12-month edoxaban group**  **(N=78)** | **3-month edoxaban group**  **(N=69)** | **Odds ratio**  **(95% CI)** | **P value** | **12-month edoxaban group**  **(N=17)** | **3-month edoxaban group**  **(N=11)** | **Odds ratio**  **(95% CI)** | **P value** |
| **Primary endpoint** |  |  |  |  |  |  |  |  |
| Symptomatic recurrent venous thromboembolism or venous thromboembolism-related death, No. (%) | 1 (1.3) | 7 (10.1) | 0.12 (0.01–0.67) | 0.013 | 0 (0) | 1 (9.1) | - | - |
| **Major secondary endpoint** |  |  |  |  |  |  |  |  |
| Major bleeding, No. (%) ^a^ | 11 (14.1) | 3 (4.3) | 3.61 (1.07–16.50) | 0.038 | 3 (17.6) | 0 (0) | - | - |
| **Other secondary endpoints** |  |  |  |  |  |  |  |  |
| Symptomatic venous thromboembolism recurrence events, No. (%) | 1 (1.3) | 7 (10.1) | 0.12 (0.01–0.67) | 0.013 | 0 (0) | 1 (9.1) | - | - |
| Venous thromboembolism-related deaths, No. (%) ^b^ | 0 (0) | 0 (0) | - | - | 0 (0) | 0 (0) | - | - |
| New or worsening thrombus images in any imaging tests during follow-up without any symptoms, No. (%) ^c^ | 11 (14.1) | 10 (14.5) | 0.97 (0.38–2.48) | 0.95 | 1 (5.9) | 1 (9.1) | 0.63 (0.02–17.00) | 0.75 |
|  |  |  |  |  |  |  |  |  |
| All clinically relevant bleeding events, No. (%) ^d^ | 21 (26.9) | 6 (8.7) | 3.87 (1.54–11.15) | 0.003 | 4 (23.5) | 1 (9.1) | 3.08 (0.38–65.30) | 0.31 |
| Deaths from all causes, No. (%) | 13 (16.7) | 16 (23.2) | 0.66 (0.29–1.50) | 0.32 | 2 (11.8) | 2 (18.2) | 0.60 (0.06–5.72) | 0.64 |

The analyses were performed for the full analysis set based on the intention-to-treat approach, which included all the patients who had undergone randomization after excluding patients who withdrew consent. For patients who did not experience an event, the time to the first event was to be censored at day 365, or the last day the patient had a complete assessment for study outcomes, whichever came first. We calculated the odds ratios, computed using the logistic regression model along with the corresponding 95% confidence intervals for all clinical endpoints, which have not been adjusted for multiple comparisons.

^a^ Major and nonmajor bleeding events were classified according to the criteria of the International Society on Thrombosis and Hemostasis.

^b^ Death due to pulmonary embolism diagnosed prior to death or at autopsy, or death unexplained by other than pulmonary embolism.

^c^ Appearance of new or worsening thrombus images in the pulmonary arteries and deep veins on imaging tests (ultrasonography of lower limb venous system, computed tomography examination, pulmonary perfusion scintigraphy, pulmonary angiography, venography) that do not match the definition of a symptomatic venous thromboembolism recurrence and are not associated with new or worsening symptoms.

^d^ For patients who had more than one event, only the first was counted.

# **Supplemental Figures**

## **Supplemental Figure 1: Time-to-event curves of the primary endpoint of symptomatic VTE recurrence or VTE-related death considering competing risk adjusted for death.**


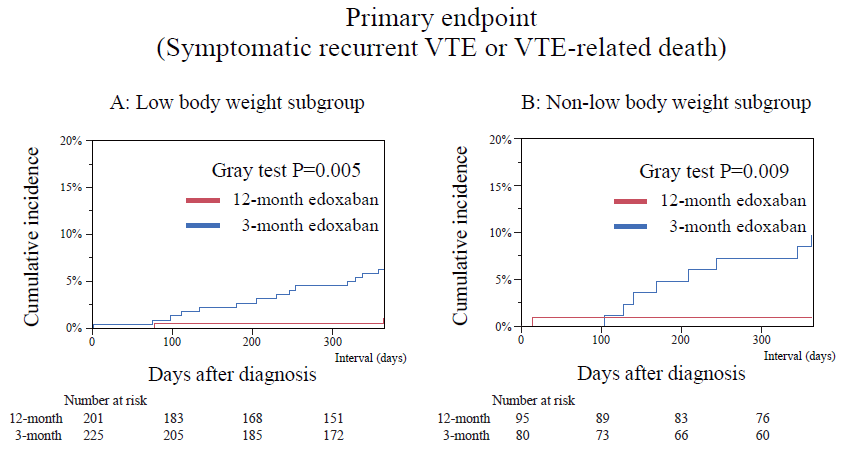


A: Low body weight subgroup. B: Non-low body weight subgroup. VTE: venous thromboembolism.

## **Supplemental Figure 2: Time-to-event curves of the major secondary endpoint of major bleeding considering competing risk adjusted for death.**


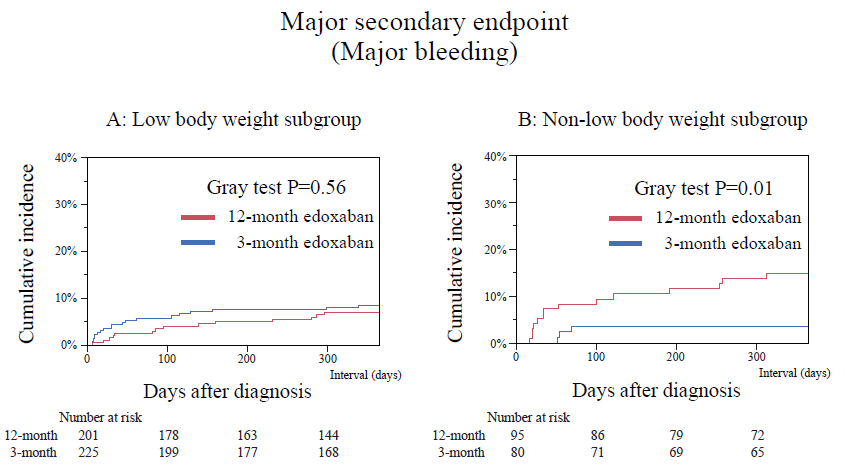


A: Low body weight subgroup. B: Non-low body weight subgroup.

## **Supplemental Figure 3: Time-to-event curves of the secondary endpoint of all clinically relevant bleeding considering competing risk adjusted for death.**


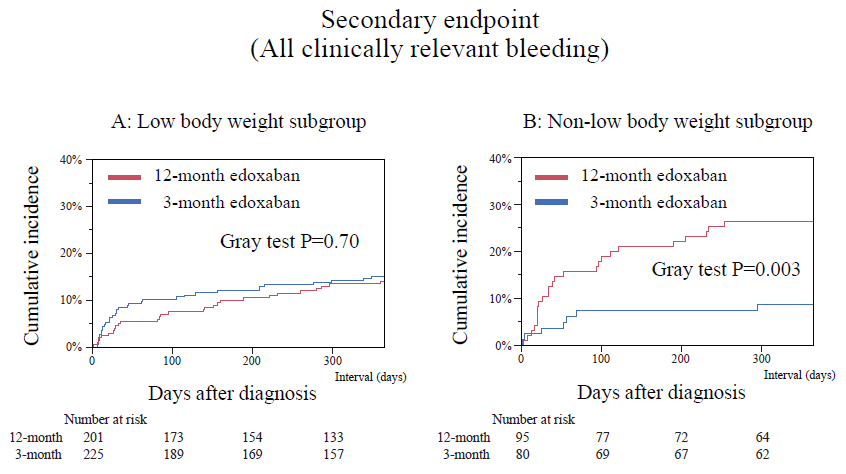


A: Low body weight subgroup. B: Non-low body weight subgroup.

## **Supplemental Figure 4: Study flowchart of per-protocol analysis**

**
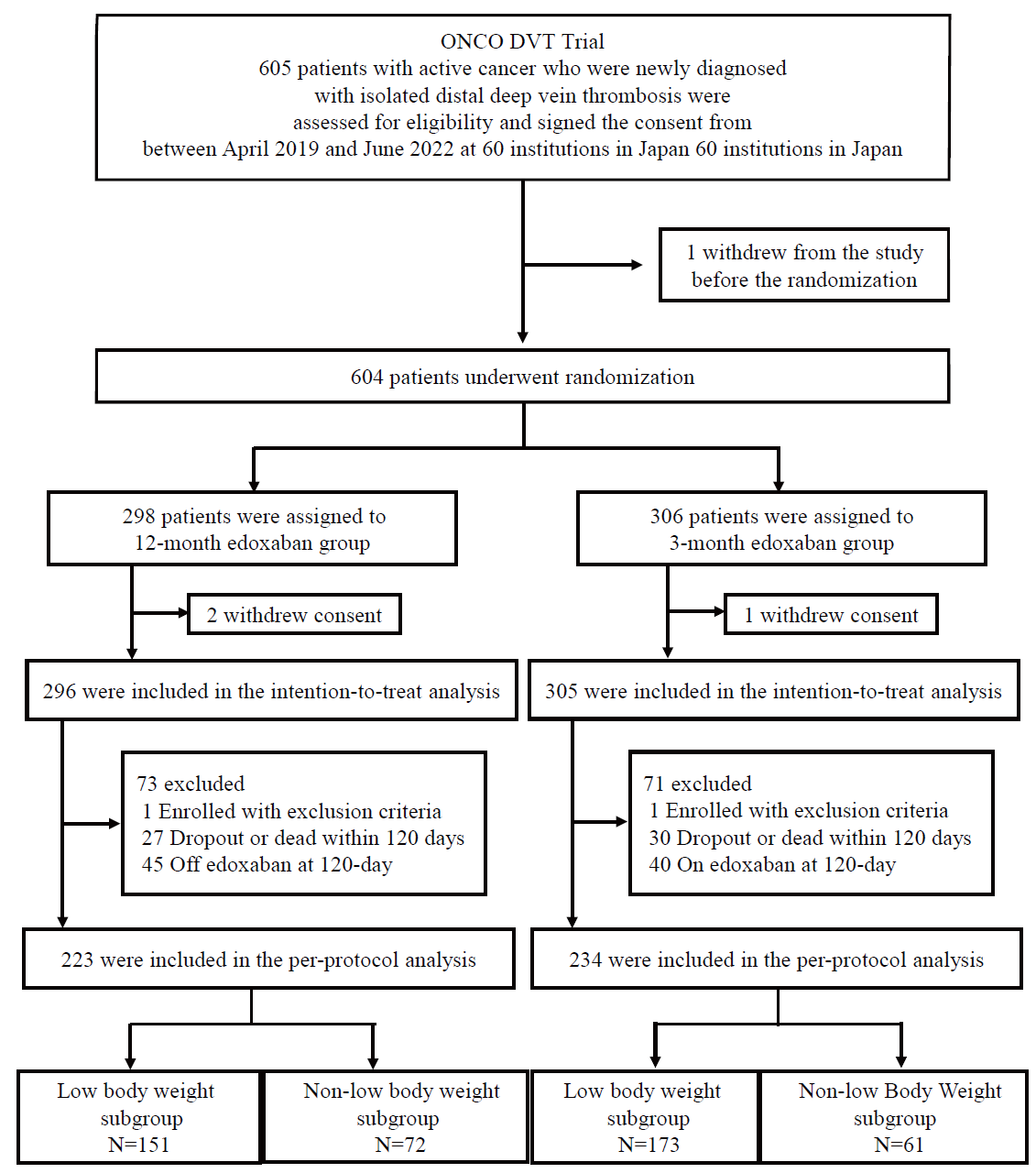
**

Low body weight was defined as a body weight ≤60 kg.

## **Supplemental Figure 5: Time-to-event curves of per-protocol analysis for persistent edoxaban discontinuation**

**
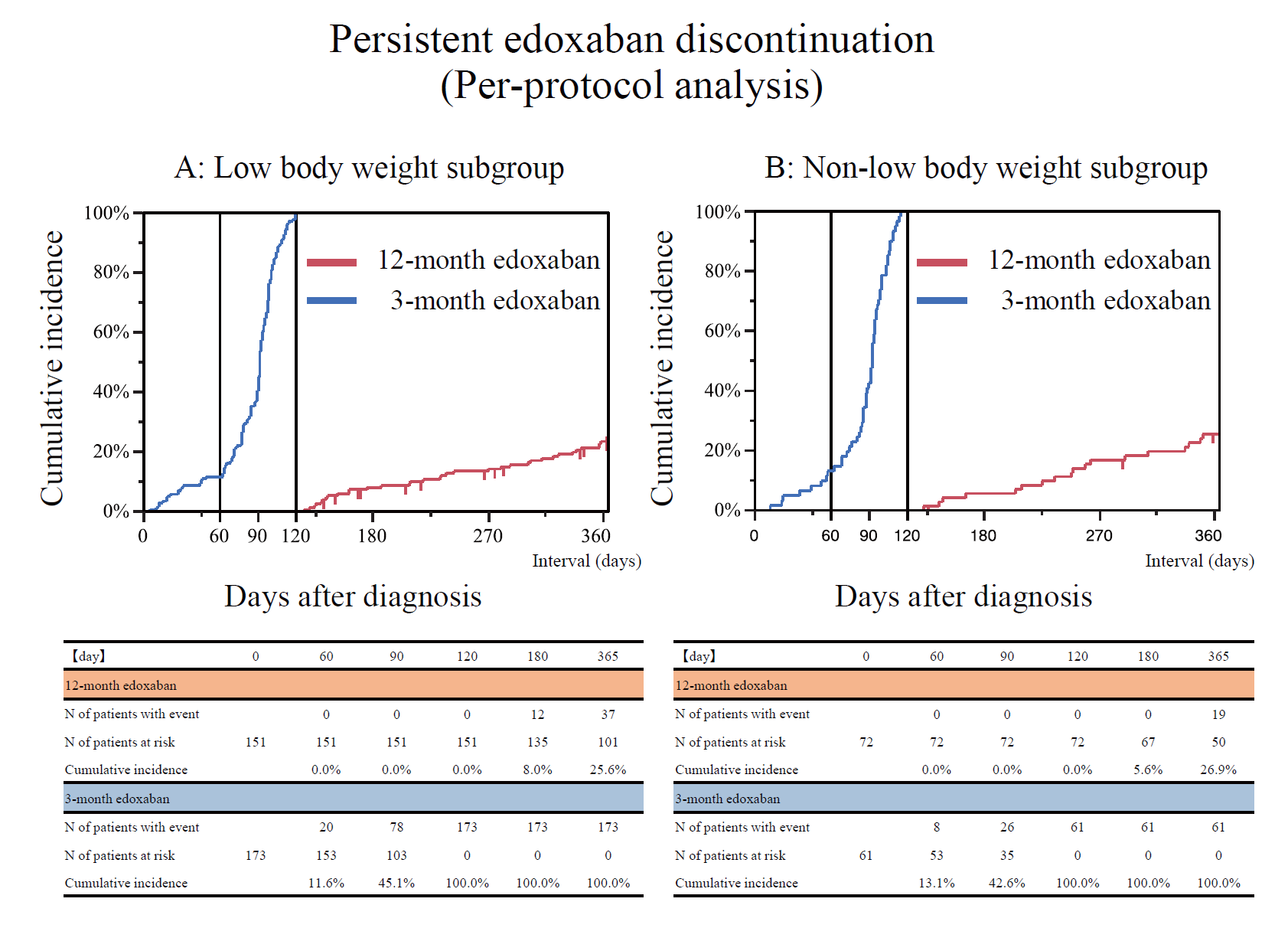
**

Persistent edoxaban discontinuation was defined as discontinuation according to the study protocol or lasting >14 days for any reason. A: Low body weight subgroup. B: Non-low body weight subgroup.

## **Supplemental Figure 6: Time-to-event curves of per-protocol analysis for the primary endpoint of symptomatic VTE recurrence or VTE-related death**

**
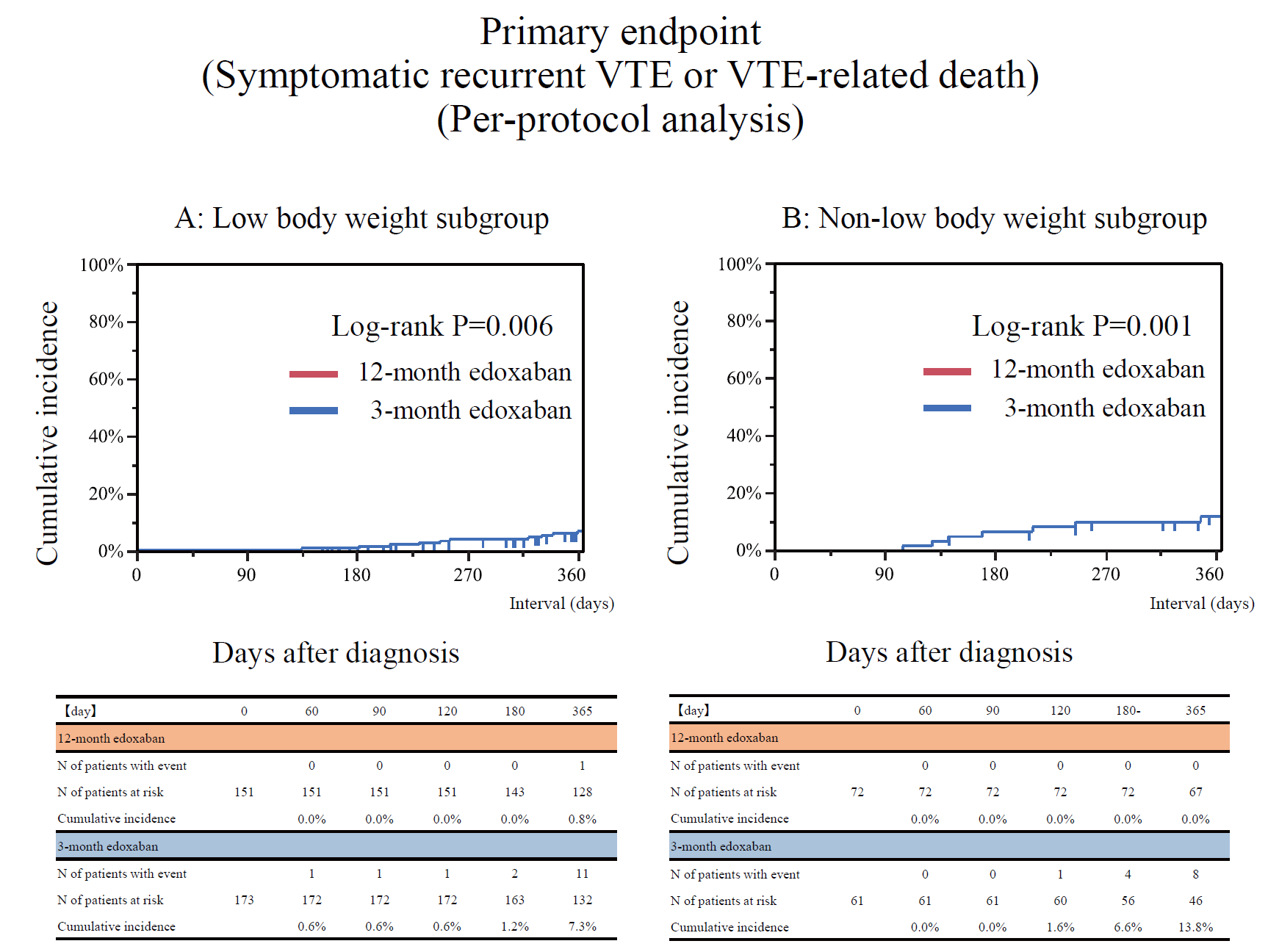
**

A: Low body weight subgroup. B: Non-low body weight subgroup. VTE: venous thromboembolism.

## **Supplemental Figure 7: Time-to-event curves of per-protocol analysis for the major secondary endpoint of major bleeding**


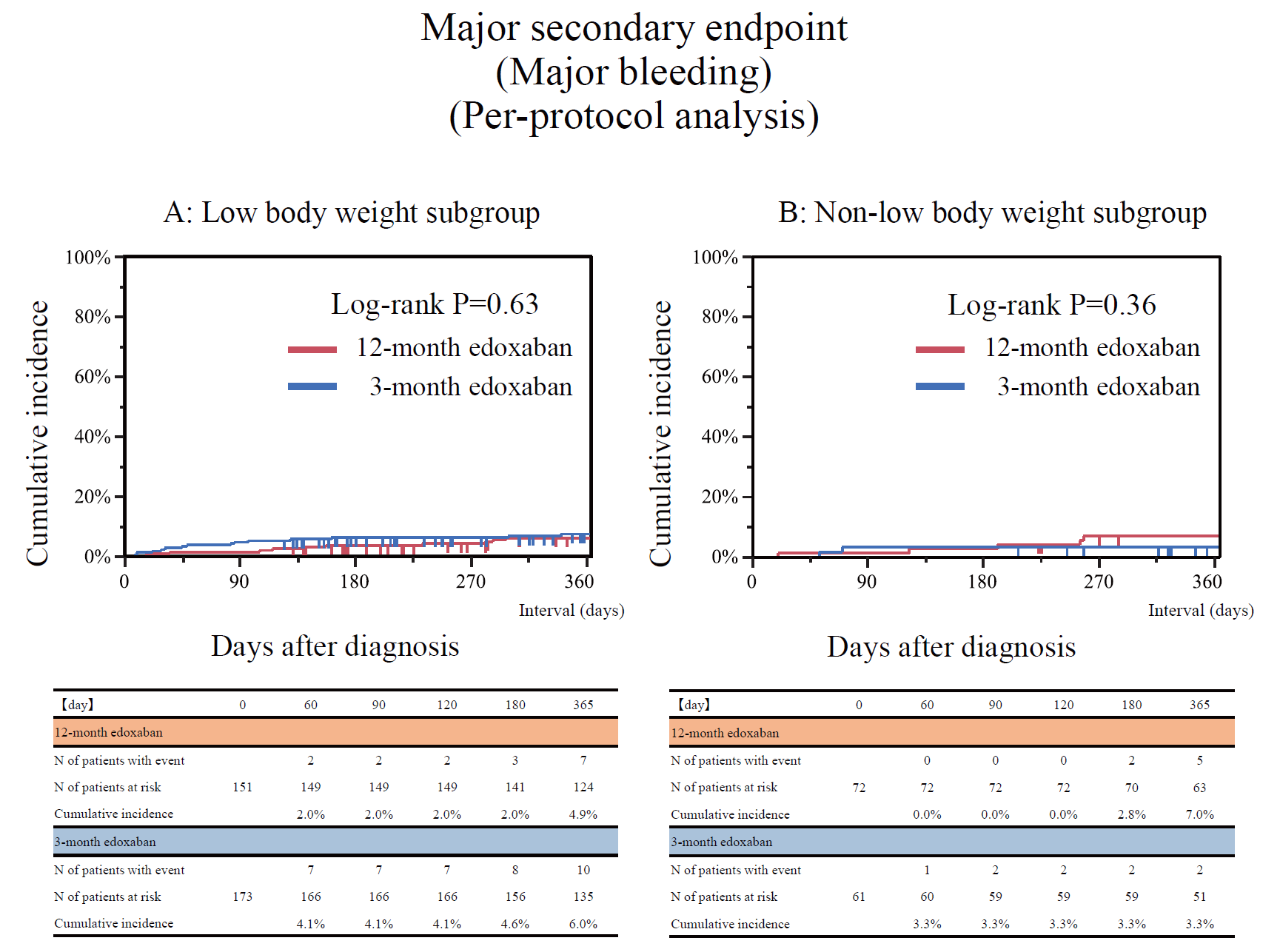


Major bleeding was defined according to International Society on Thrombosis and Haemostasis criteria. A: Low body weight subgroup. B: Non-low body weight subgroup.

## **Supplemental Figure 8: Time-to-event curves of per-protocol analysis for the secondary endpoint of all clinically relevant bleeding**


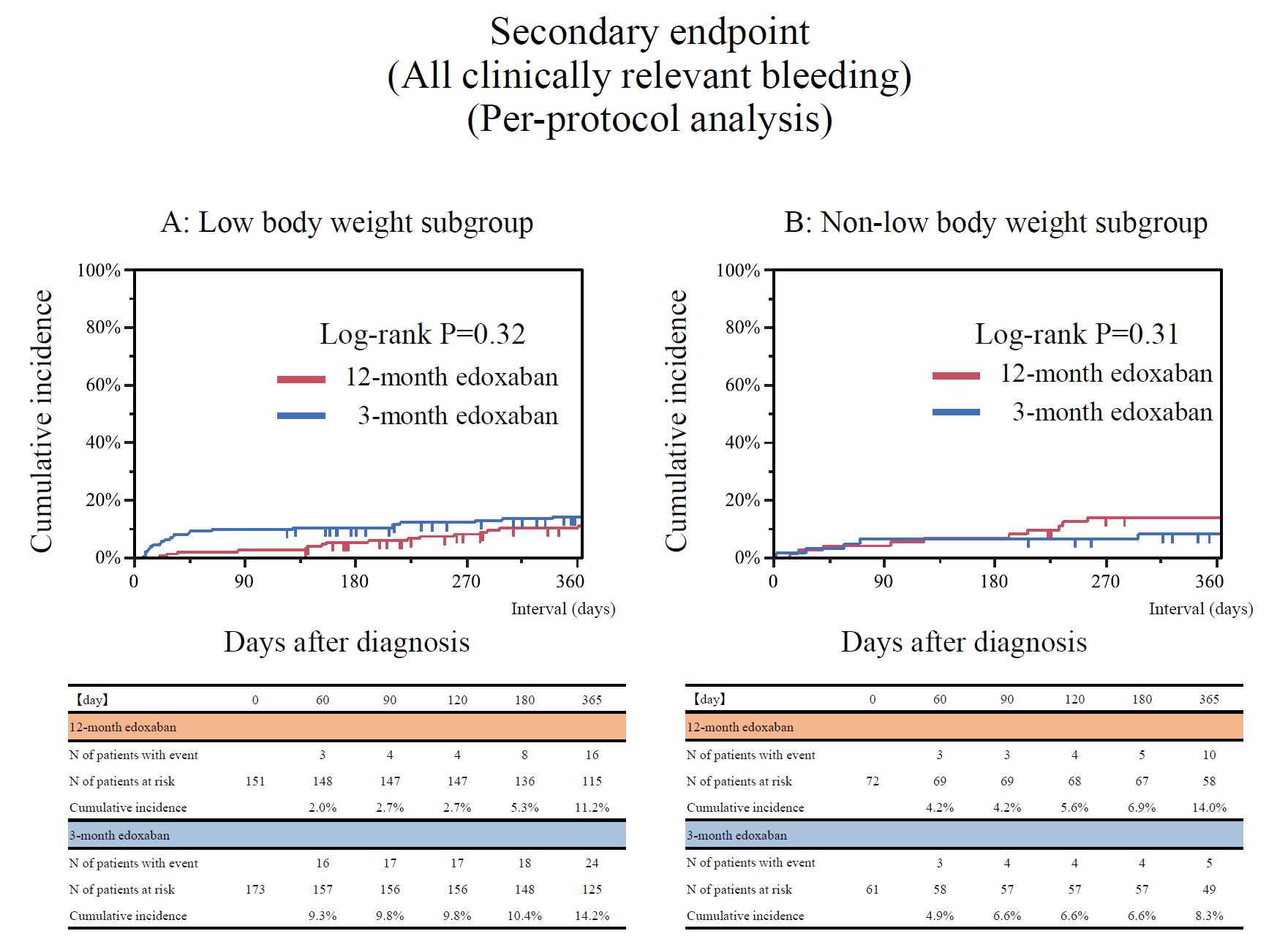


A: Low body weight subgroup. B: Non-low body weight subgroup.

## **Supplemental Figure 9: Study flowchart of as-treated analysis**


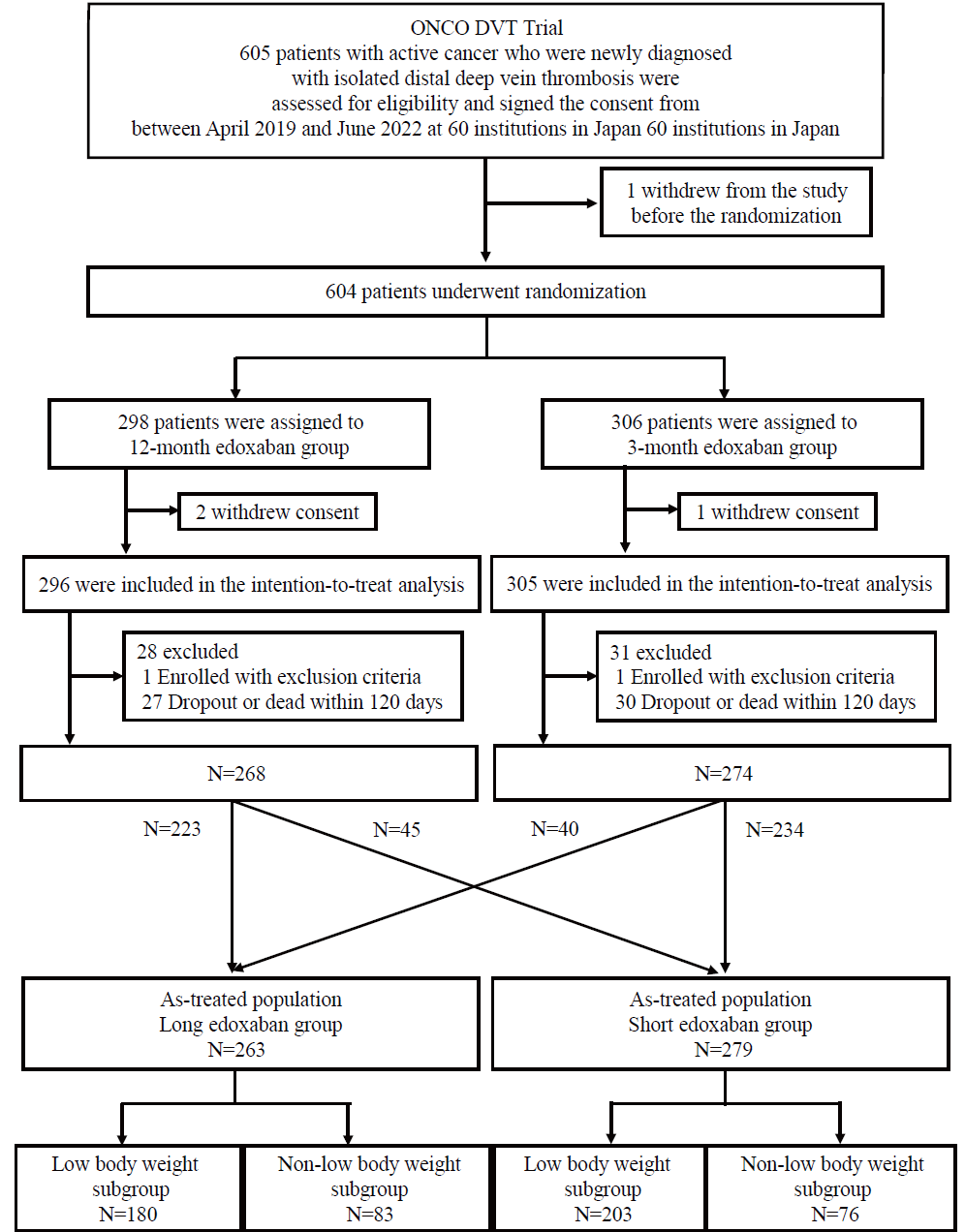


Low body weight was defined as a body weight ≤60 kg.

## **Supplemental Figure 10: Time-to-event curves of as-treated analysis for persistent edoxaban discontinuation**


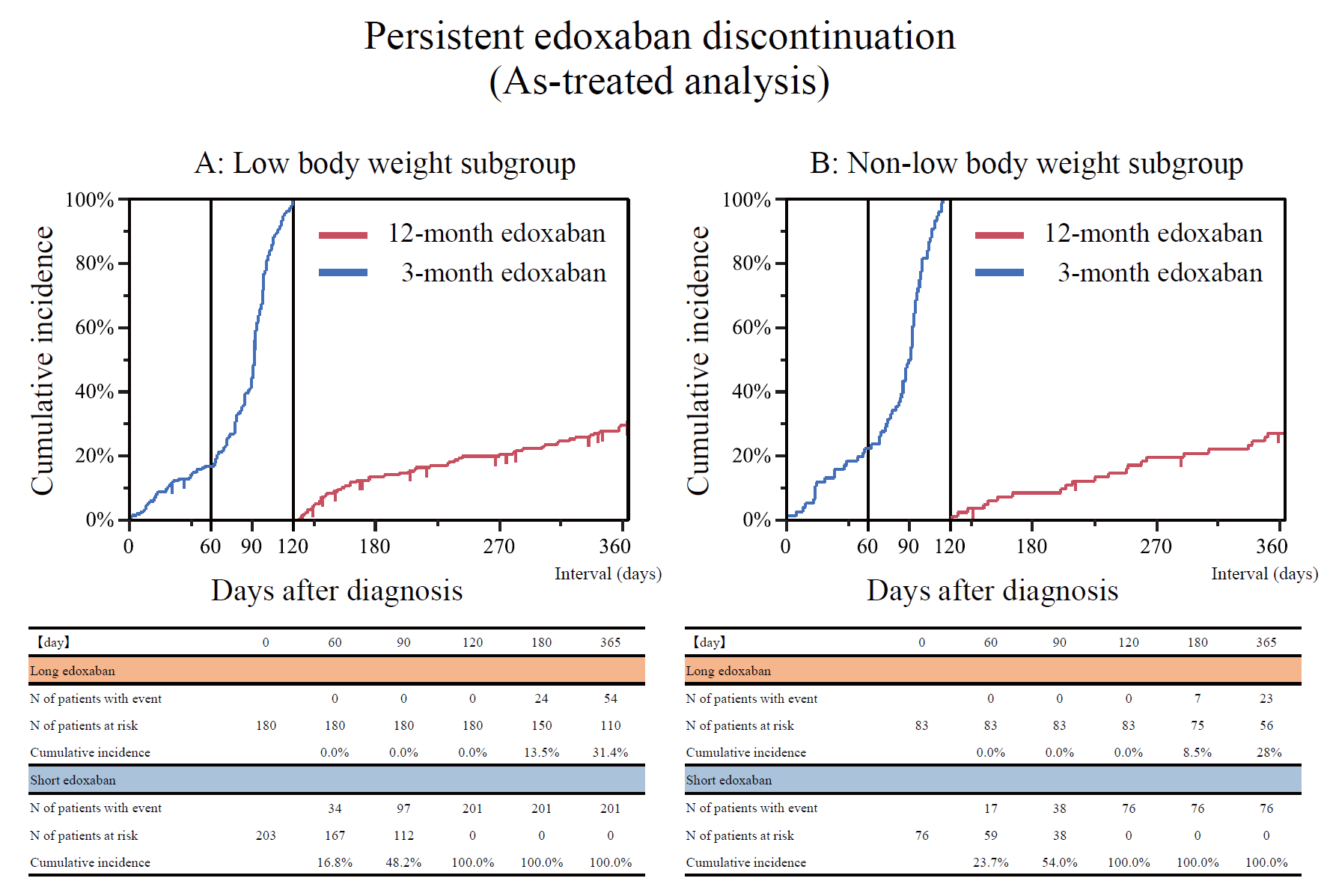


Persistent edoxaban discontinuation was defined as discontinuation according to the study protocol or lasting >14 days for any reason. A: Low body weight subgroup. B: Non-low body weight subgroup.

## **Supplemental Figure 11: Time-to-event curves of as-treated analysis for the primary endpoint of symptomatic VTE recurrence or VTE-related death**


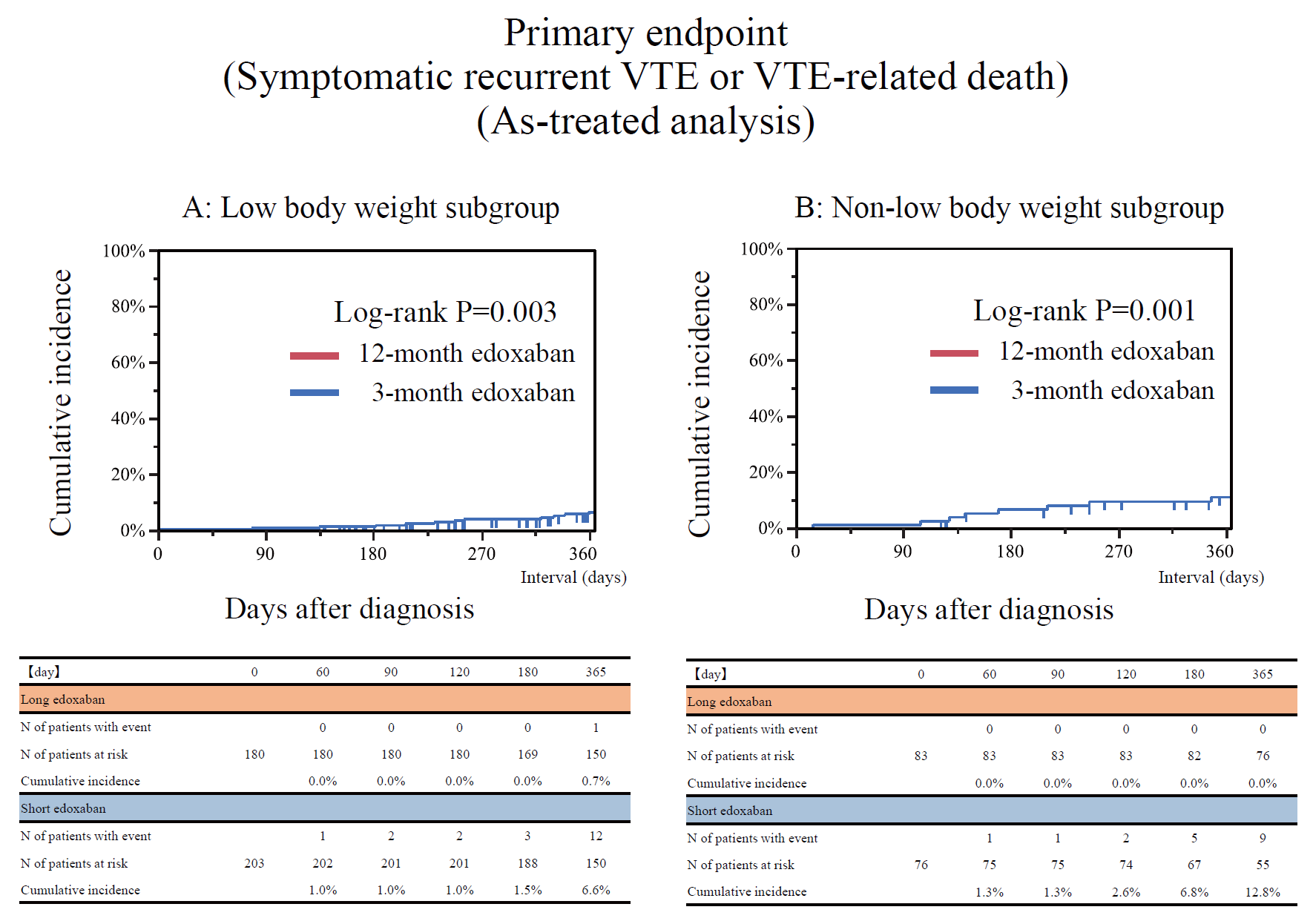


A: Low body weight subgroup. B: Non-low body weight subgroup. VTE: venous thromboembolism.

## **Supplemental Figure 12: Time-to-event curves of as-treated analysis for the major secondary endpoint of major bleeding**


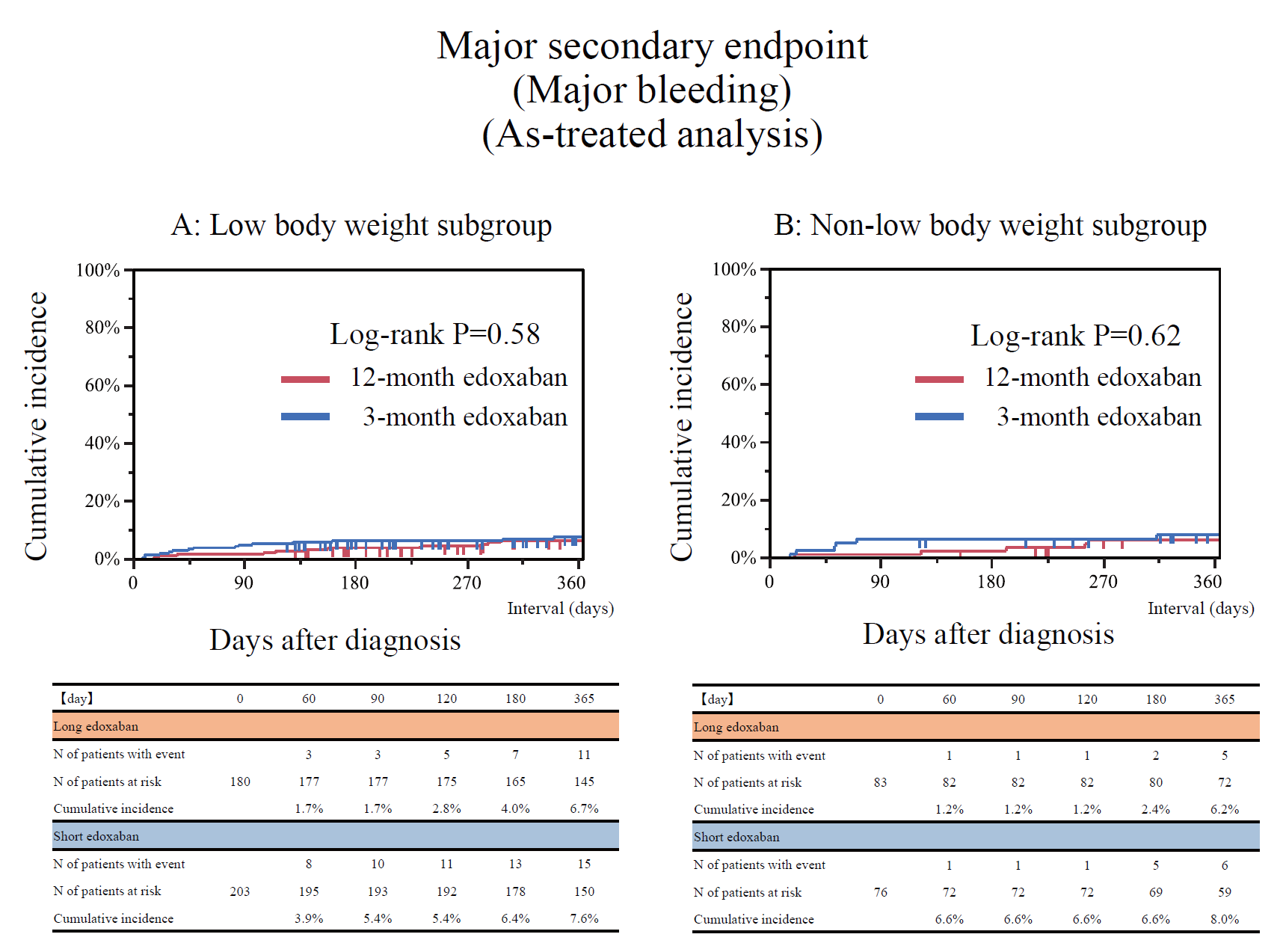


Major bleeding was defined according to International Society on Thrombosis and Haemostasis criteria. A: Low body weight subgroup. B: Non-low body weight subgroup.

## **Supplemental Figure 13: Time-to-event curves of as-treated analysis for the secondary endpoint of all clinically relevant bleeding**


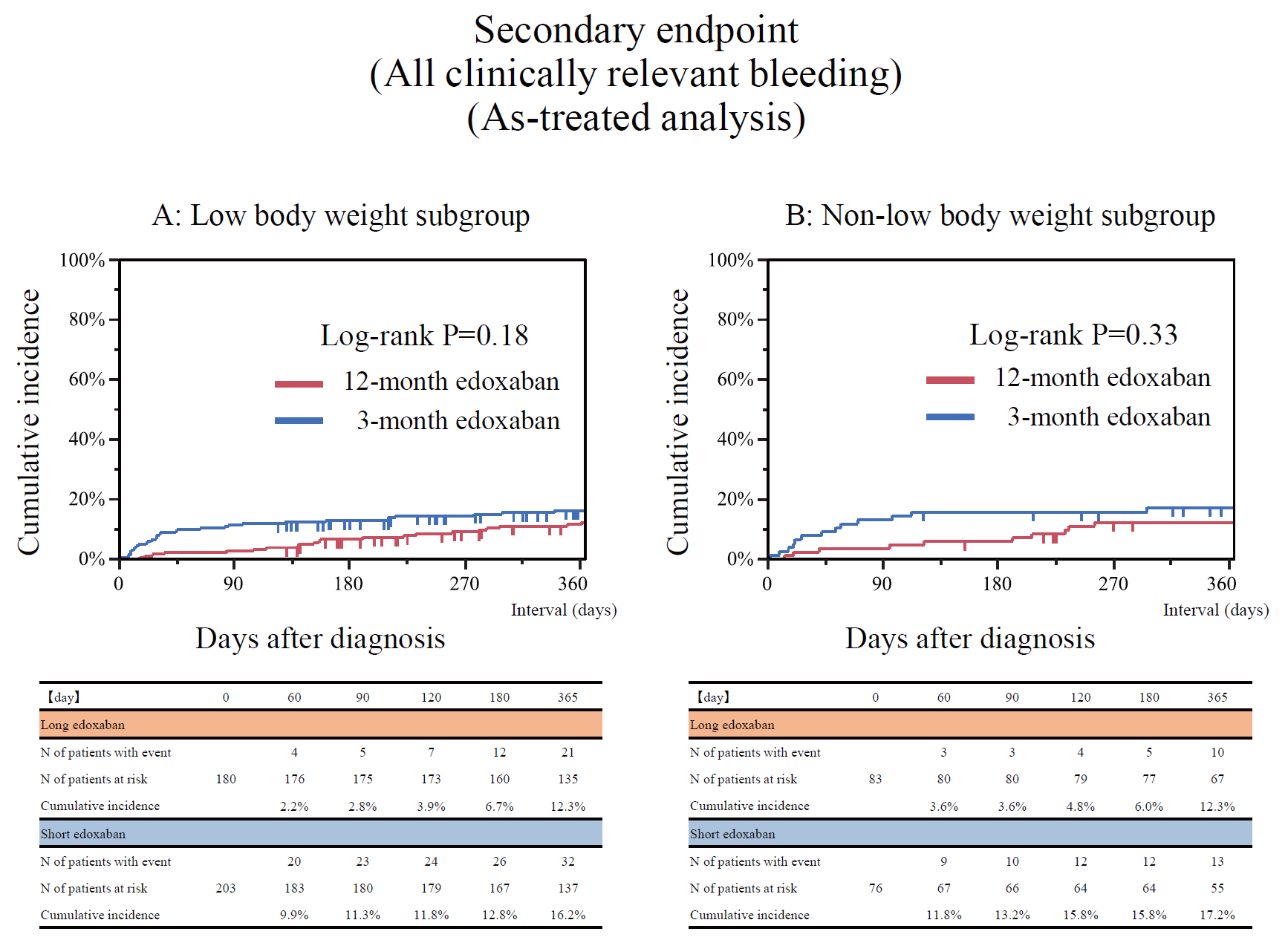


A: Low body weight subgroup. B: Non-low body weight subgroup.

## **Supplemental Figure 14: Time-to-event curves of persistent edoxaban discontinuation according to the doses of edoxaban in the non-low body weight subgroup**


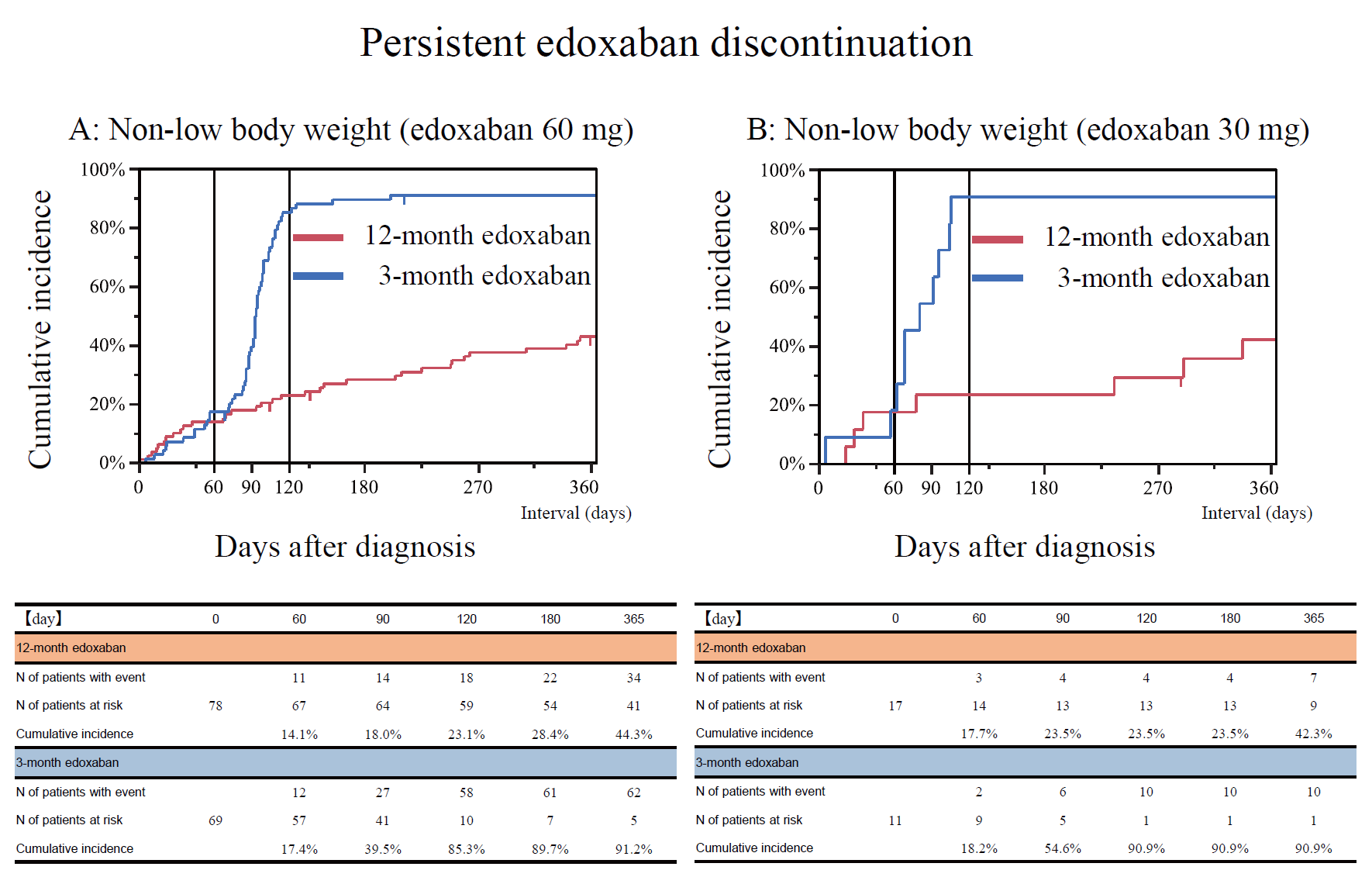


Persistent edoxaban discontinuation was defined as discontinuation according to the study protocol or lasting >14 days for any reason. A: Patients with standard edoxaban treatment of 60 mg daily. B: Patients with reduced edoxaban treatment of 30 mg daily.

## **Supplemental Figure 15: Time-to-event curves of primary endpoint of symptomatic VTE recurrence or VTE-related death according to the doses of edoxaban in the non-low body weight subgroup**


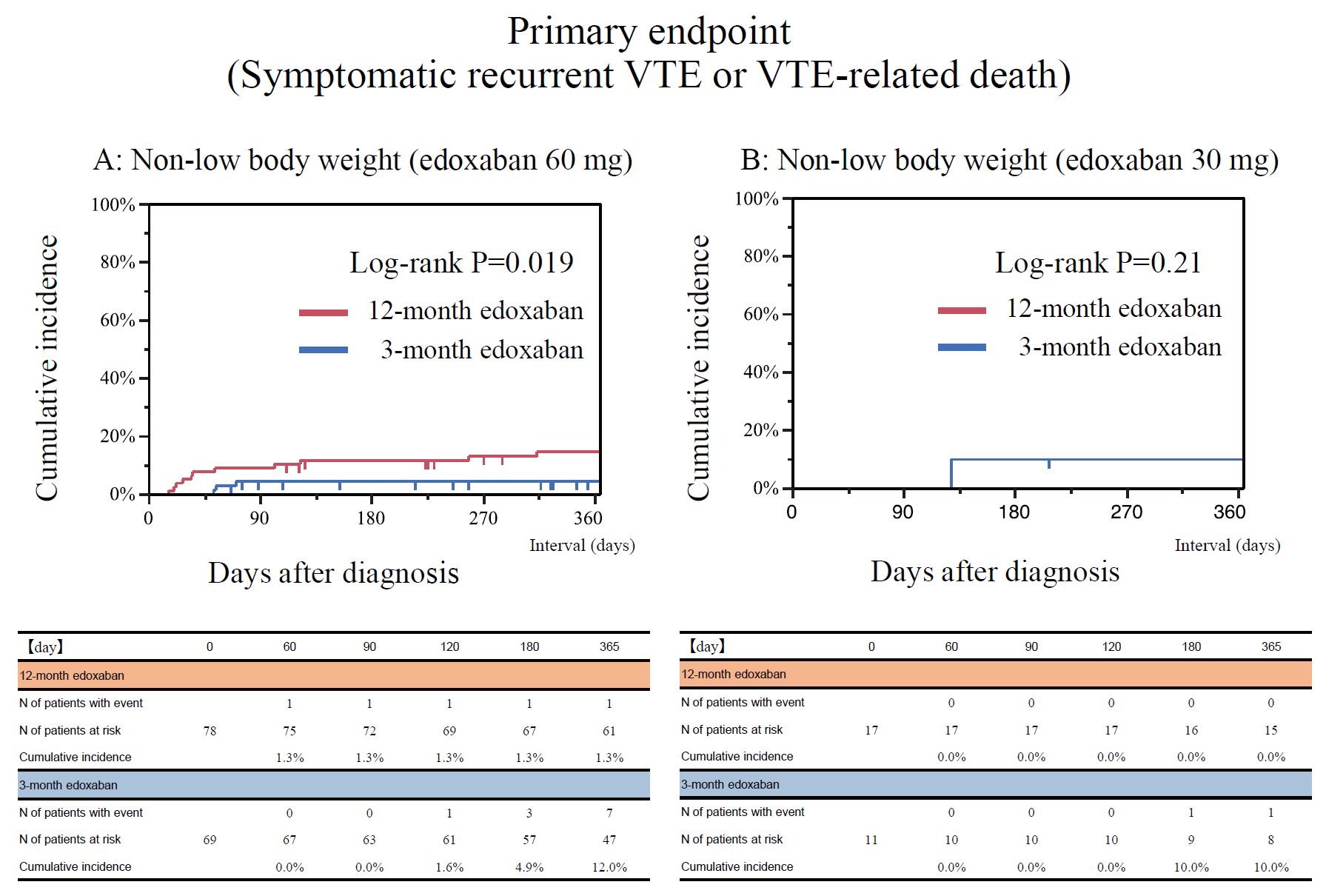


A: Patients with standard edoxaban treatment of 60 mg daily. B: Patients with reduced edoxaban treatment of 30 mg daily. VTE: venous thromboembolism.

## **Supplemental Figure 16: Time-to-event curves of major secondary endpoint of major bleeding according to the doses of edoxaban in the non-low body weight subgroup**


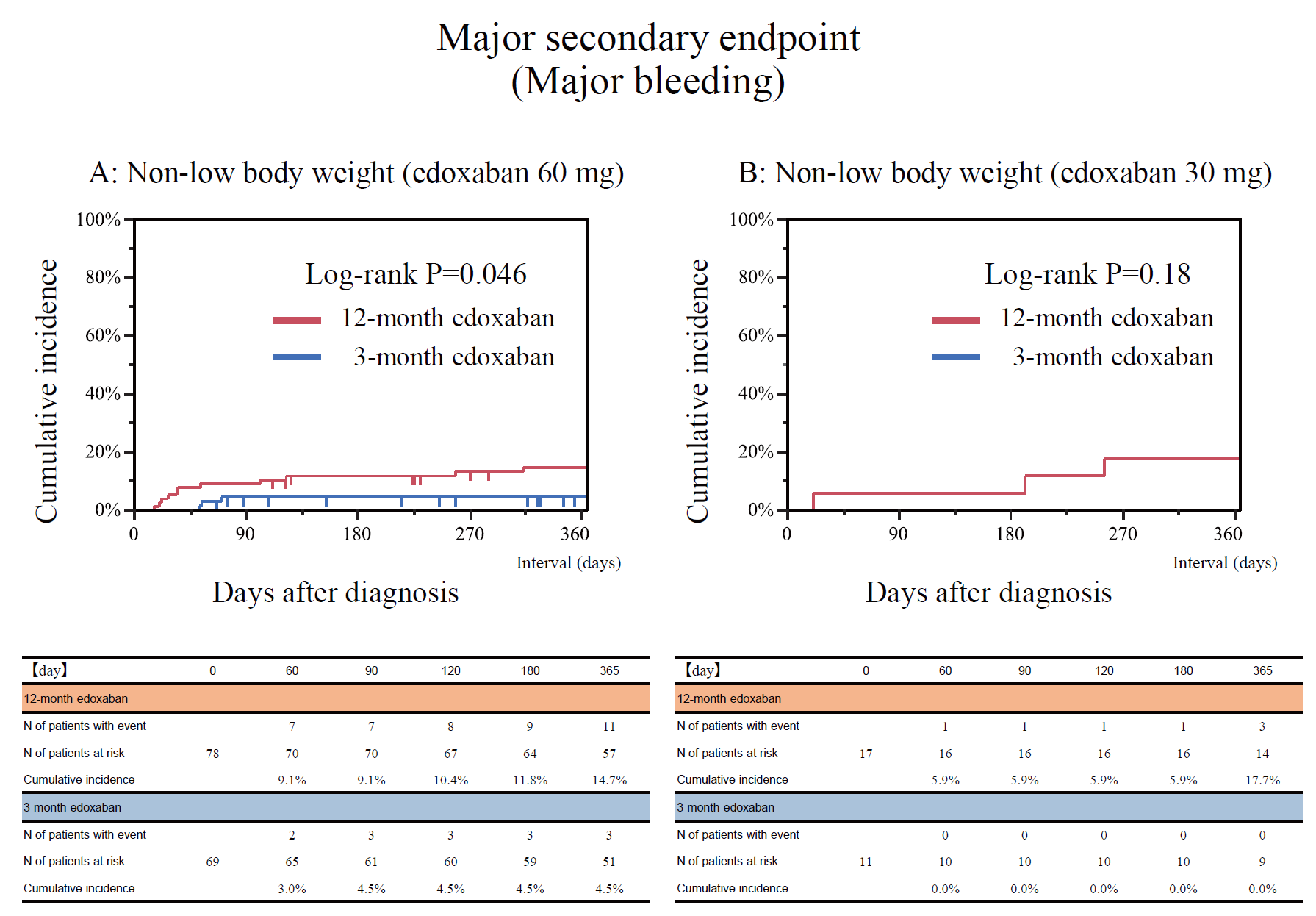


Major bleeding was defined according to International Society on Thrombosis and Haemostasis criteria. A: Patients with standard edoxaban treatment of 60 mg daily. B: Patients with reduced edoxaban treatment of 30 mg daily.

## **Supplemental Figure 17: Time-to-event curves of secondary endpoint of all clinically relevant bleeding according to the doses of edoxaban in the non-low body weight subgroup**


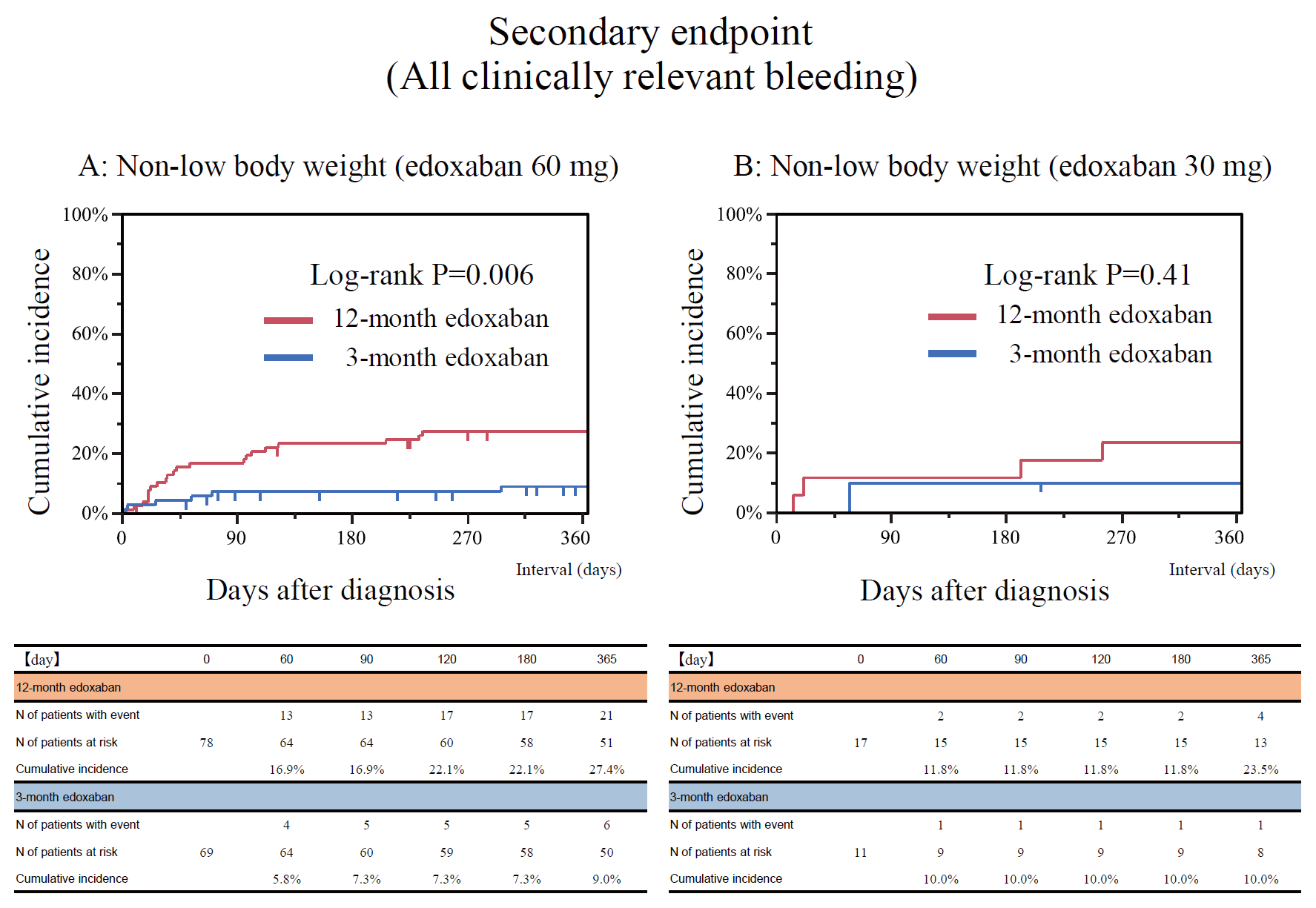


A: Patients with standard edoxaban treatment of 60 mg daily. B: Patients with reduced edoxaban treatment of 30 mg daily.
